# Effects of Foliar Selenium Treatment on Phenolic Antioxidant Compound Accumulation and Transcriptomic Profiles in Developing Black Wheat Grains

**DOI:** 10.3390/plants15182880

**Published:** 2026-09-20

**Authors:** Tianci Shen, Binyan Yuan, Qingsong Ba, Lanlan Zhang, Pengfei Zhang, Gensheng Zhang, Guiping Li, Yue Zhuo

**Affiliations:** 1 Huaibei Key Laboratory of Crop Genetic Improvement and Efficient Green Safe Production, Huaibei 235000, China; 2 Anhui Province Key Laboratory of Pollutant Sensitive Materials and Environmental Remediation, Huaibei 235000, China; 3 College of Life Sciences, Huaibei Normal University, Huaibei 235000, China; 4 Xiangyang Academy of Agricultural Sciences, Xiangyang 441057, China; 5 Suixi County Agricultural Research Experiment Station, Huaibei 235000, China

**Keywords:** *Triticum aestivum* L., selenium, flavonoid biosynthesis, phenylpropanoid pathway, transcriptomic profiling, *TaMYB20*-like, *CRISPR/Cas*9

## Abstract

Selenium (Se) foliar spraying improves grain nutritional quality of pigmented wheat, yet the physiological and molecular regulatory mechanisms of Se-mediated anthocyanin accumulation in black wheat remain poorly understood. In this study, six concentrations of sodium selenite (0–150 mg·L^−1^, 0 mg·L^−1^ served as the blank control) were foliarly applied to black wheat Nongda 3753 and white wheat Jimai 22 to screen the optimal Se dosage. Dynamic changes in grain antioxidants across multiple time points throughout grain filling were characterized, and transcriptome sequencing combined with an Arabidopsis mutant functional assay was performed to dissect the regulatory network. The results showed that grain Se content in Nongda 3753 reached its peak (0.61 mg·kg^−1^) under the 150 mg·L^−1^ treatment, representing a 555.63% increase over the control. Under the 75 mg·L^−1^ treatment, total phenolics (2.68 mg GAE·g^−1^), total flavonoids (2.96 mg rutin·g^−1^), and DPPH scavenging rate (89.02%) all reached their maximum levels; therefore, 75 mg·L^−1^ was determined as the optimal compromise dosage for comprehensive grain quality improvement. Through temporal profiling, 25 days after anthesis (DAA) was identified as the critical Se-responsive stage, at which anthocyanin content reached its highest level among all time points (117.08 mg·kg^−1^), showing a significant increase of 25.13% compared with the control at the same time point, and the anthocyanin biosynthesis pathway was uniquely enriched. The anthocyanidin synthase (*ANS*) gene was specifically upregulated by Se, and nine key transcription factors were screened. Functional verification revealed that TaMYB20-like acts as a negative regulator of anthocyanin accumulation. This work clarifies the temporal pattern and molecular basis of Se-enhanced antioxidant synthesis, offering gene targets for Se-enriched functional wheat breeding.

## 1. Introduction

Wheat (*Triticum aestivum* L.) is one of the most important staple crops globally, supplying approximately 20% of dietary caloric and protein for global human populations [1]. With continuous improvements in living standards, consumer demand for wheat products has shifted from basic calorie supply toward functional foods with health-promoting bioactive ingredients [2]. Against this backdrop, colored wheat varieties, especially black wheat, have drawn extensive research attention due to their abundant grain anthocyanin reserves [3,4]. The pericarp and aleurone layers of black wheat grains accumulate abundant anthocyanin pigments, resulting in far higher anthocyanin, total phenolic and total flavonoid concentrations compared with purple, blue and ordinary white wheat grains [5,6]. Multiple animal model experiments have indicated that long-term intake of black wheat extracts can alleviate metabolic disorders, regulate intestinal microflora homeostasis and reduce blood glucose levels [7,8]. Despite the well-documented nutritional advantages of black wheat, few systematic studies have investigated agronomic strategies to elevate grain antioxidant levels or deciphered their underlying molecular regulatory mechanisms.

Selenium (Se) is an essential micronutrient for humans, participating in critical physiological processes, including antioxidant defense, immune regulation, and thyroid hormone metabolism [9,10]. It is estimated that 0.5–1 billion people globally suffer from insufficient Se dietary intake, and wheat grains constitute a major natural Se source for human populations [11,12]. Foliar application of Se fertilizers is an efficient agronomic biofortification strategy for increasing grain Se concentration [13,14]. Previous investigations have confirmed that appropriate exogenous Se application can activate core structural genes of the phenylpropanoid pathway (e.g., *PAL*, *CHS*, *ANS*), thereby promoting the biosynthesis of phenolic acids, flavonoids and anthocyanins in cereal grains [15,16]. This dual regulatory property of Se creates a promising technical route to simultaneously enhance grain Se enrichment and the antioxidant quality of black wheat. However, Se displays a typical hormetic effect on plant secondary metabolism: low concentrations promote metabolite biosynthesis, whereas high concentrations induce growth suppression, and the response intensity differs significantly among wheat genotypes with distinct grain pigmentation [15]. Notably, pigmented wheat genotypes have inherently activated flavonoid and anthocyanin biosynthetic pathways in grains, rendering them more sensitive to exogenous elicitors, including selenium, while anthocyanin biosynthesis is largely silenced in grains of white wheat varieties. The stronger intrinsic antioxidant defense system in black wheat can mitigate the oxidative stress triggered by selenium absorption, which in turn supports continuous selenium assimilation and accumulation, thereby conferring black wheat with higher potential for enriching selenium and phenolic antioxidants than white wheat.

Selenium exhibits multifaceted and species-dependent effects on growth, mineral accumulation and secondary metabolism across diverse crop species. Apart from cereal crops such as wheat, investigations on horticultural crops also reveal that different selenium forms can modulate seedling vigor and reshape tissue mineral profiles, which highlights the broad application prospect of selenium-based agronomic biofortification strategies across plant taxa [17].

In addition, existing relevant studies mostly detect only mature grain phenotypes and metabolites and lack continuous dynamic monitoring of antioxidant accumulation throughout grain filling. Therefore, screening the optimal foliar Se dosage for black wheat and clarifying temporal metabolite variation patterns during grain filling are necessary prerequisites for subsequent molecular mechanistic research.

Grain filling is a critical period for the biosynthesis and accumulation of antioxidant substances. Anthocyanin biosynthesis, a major branch of the phenylpropanoid pathway, is catalyzed by a series of well-characterized structural genes, including *PAL*, *4CL*, *CHS*, *CHI*, *F3H*, *F3′H*, *DFR*, and *ANS*, with the final products being glycosylated by *UF3GT*/*BZ1* and transported into the vacuole by GST [18,19]. At the transcriptional level, the MBW (MYB-bHLH-WD40) ternary complex dominates anthocyanin pathway transcription, among which R2R3-MYB transcription factors determine tissue-specific and stage-specific pigment accumulation characteristics [20,21]. Previous research on purple wheat demonstrated that grain anthocyanin levels peak at 25–30 days after anthesis (DAA) [22]. Nevertheless, whether exogenous Se reshapes the temporal accumulation curve of anthocyanins in black wheat remains unknown, and the complete transcriptional regulatory cascade remains unreported. Four core scientific questions require clarification: (1) How does foliar Se spraying alter anthocyanin biosynthesis dynamics during black wheat grain filling? (2) Which structural genes mediate Se-triggered pigment accumulation? (3) What transcription factors participate in Se signal transduction to regulate flavonoid metabolism? (4) What is the genome-wide transcriptional landscape of black wheat grains responding to Se treatment?

Nevertheless, the upstream selenium-sensing signaling cascade mediating the transcriptional repression of MYB20-like homologs remains largely uncharacterized in cereal crops. Whether MYB20-like transcription factors directly bind to the promoters of anthocyanin biosynthetic structural genes (*ANS*, *DFR*, *CHS*) also awaits further biochemical validation, which represents a critical knowledge gap for understanding selenium-driven flavonoid regulation in wheat.

To fill the above research gaps, the present study was carried out with four specific objectives: (1) identify the optimal foliar sodium selenite concentration for simultaneous Se and anthocyanin enrichment in black wheat; (2) characterize the dynamic accumulation patterns of antioxidant metabolites across four grain-filling stages; (3) reveal the Se-responsive transcriptional network of phenylpropanoid and anthocyanin pathways via transcriptome analysis; (4) preliminarily validate the repressive function of the candidate *TaMYB20-like* using Arabidopsis *AtMYB20* CRISPR mutants.

## 2. Materials and Methods

### 2.1. Plant Materials and Experimental Design

Field trials were carried out during the 2023–2024 wheat growing season at the experimental station in Huaibei City, Anhui Province, China. Two wheat genotypes were selected: black-grained cultivar Nongda 3753 and common white-grained cultivar Jimai 22. Six sodium selenite (Na_2_SeO_3_) concentrations were applied: 0 (blank control, CK), 50, 75, 100, 125, and 150 mg·L^−1^. The experiment adopted a split-plot design, with wheat genotype as the main plot factor and Se concentration as the subplot factor; each treatment contained three independent biological replicates, with a plot size of 4 m^2^ (2 m × 2 m), arranged in a randomized complete block design. Foliar spraying was initiated at full anthesis (50% spike flowering), repeated once every seven days for three consecutive applications. All foliar spray treatments (sodium selenite solution and distilled-water control) were applied in late afternoon under ambient field temperatures of 22–26 °C, avoiding periods of high temperature at noon. The Se solution was applied at a volume of 750 L·ha^−1^ per application (corresponding to 300 mL per 4 m^2^ plot). Tween-20 was added as a surfactant at a final concentration of 0.1% (*v*/*v*) to ensure uniform droplet adhesion to and efficient foliar absorption by the spikes and flag leaves. The control group was sprayed with equal volumes of distilled water. All field cultivation and pest management practices followed local standard wheat production protocols.

### 2.2. Sample Collection

At the grain-filling stage, 100 uniform, synchronously flowering main spikes were tagged in each subplot. Grain samples were harvested at 5, 15, 25, and 35 DAA (physiological maturity stage). For each sampling time point, 20–30 tagged spikes were randomly collected per plot and manually threshed. Fresh grain samples were immediately frozen in liquid nitrogen and transferred to −80 °C ultra-low temperature refrigerators for long-term storage. Three independent plot replicates were retained for all biochemical and molecular detection experiments.

### 2.3. Determination of Physiological and Biochemical Parameters

Grain total selenium concentration was quantified by hydride generation atomic fluorescence spectrometry (HG-AFS), following the first method described in the Chinese national food-safety standard GB 5009.93-2017 [23]. Briefly, grain samples were digested by acid heating; hexavalent selenium was reduced to tetravalent selenium under hydrochloric-acid medium. Selenium hydride gas was generated using sodium borohydride as reductant and transported by carrier gas into the atomizer for atomic-fluorescence measurement. This method avoids 2,3-diaminonaphthalene solvent-extraction procedures required for molecular fluorometric assays [13].

The anthocyanin content was measured by the pH differential method [24]. Absorbance values at 510 nm and 700 nm were recorded, and the anthocyanin content was calculated as cyanidin-3-glucoside equivalents using the formula: anthocyanin content (mg·kg^−1^) = (A × MW × DF × V)/(ε × m), where A = (A_510_ − A_700_)pH_1.0_ − (A_510_ − A_700_)pH_4.5_, MW = 449.2 g·mol^−1^, and ε = 26,900 L·mol^−1^·cm^−1^. Total phenolic content was determined by the Folin–Ciocalteu method [25], with absorbance detected at 765 nm and results expressed as gallic acid equivalents (mg GAE·g^−1^). Total flavonoid content was measured using the aluminum chloride chromogenic reaction at 510 nm, expressed as rutin equivalents (mg rutin·g^−1^) [26]. Three complementary antioxidant activity assays were conducted: DPPH radical scavenging capacity (517 nm) [27], FRAP reducing power (593 nm) [28], and ABTS radical scavenging activity (734 nm) [29].

### 2.4. RNA Extraction, Library Construction, and Sequencing

Total RNA was extracted from frozen grain samples using the Trizol reagent method [30]. All RNA-seq libraries were constructed from the black wheat cultivar Nongda 3753. cDNA libraries were constructed using the Hieff NGS^TM^ MaxUp mRNA Library Prep Kit and sequenced on an Illumina NovaSeq 6000 platform with 150 bp paired-end reads. A total of 24 libraries were constructed: two treatments (SeT and CK) × four stages (5, 15, 25, and 35 DAA) × three biological replicates.

### 2.5. Sequencing Data Processing and Bioinformatics Analysis

Raw reads were quality-filtered using Trimmomatic v0.36. Clean reads were aligned to the wheat reference genome (IWGSC RefSeq v2.1) with HISAT2 v2.1.0, and gene expression levels were quantified as transcripts per million (TPM) using StringTie v1.3.3b. Differentially expressed genes (DEGs) were identified with DESeq2 v1.12.4 (|log_2_(FC)| ≥ 1, FDR < 0.05). GO and KEGG enrichment analyses were performed using topGO v2.24.0 and clusterProfiler v3.0.5, respectively (q-value < 0.05).

### 2.6. RT-qPCR Validation

Nine DEGs were randomly selected for RT-qPCR validation. RT-qPCR was performed on a Roche LightCycler 96 system (Roche Life Science, Germany) using NovoStart^®^ SYBR High-Sensitivity qPCR SuperMix (Novoprotein, E009). Each 10 µL reaction contained 5 µL of 2× SYBR SuperMix, 0.4 µL each of forward and reverse primers (10 µmol·L^−1^), 1 µL of cDNA template, and 3.2 µL of nuclease-free water. The thermal cycling protocol was as follows: initial denaturation at 95 °C for 60 s, followed by 40 cycles of 95 °C for 10 s, 60 °C for 20 s, and 72 °C for 30 s. A melting curve analysis was performed to confirm amplicon specificity. The wheat Actin gene (*TaActin*) served as the internal reference, and relative expression levels were calculated using the 2^−ΔΔCt^ method [31]. Primer sequences are provided in Supplementary Table S1.

### 2.7. Bioinformatics Analysis of Tamyb20-like

Conserved domains, physicochemical properties, phosphorylation sites, signal peptides, transmembrane domains, and secondary and tertiary protein structures of TaMYB20-like were predicted using SMART, ProtParam, ProtScale, NetPhos-3.1, SignalP-5.0, TMHMM-2.0, SOPMA, and SWISS-MODEL, respectively. MYB homologous protein sequences were retrieved from NCBI and TAIR databases. A neighbor-joining phylogenetic tree with 1000 bootstrap replicates was constructed using MEGA X software.

### 2.8. Functional Validation Using the Arabidopsis Atmyb20 Mutant

Homozygous AtMYB20 CRISPR/Cas9 mutant Arabidopsis thaliana lines were purchased from Biogene Biotech (Suzhou, China), with Col-0 wild-type as the control. The mutant and wild-type plants were grown in a controlled growth chamber under a 16 h light/8 h dark photoperiod, light intensity of 120 µmol·m^−2^·s^−1^, temperature of 22 ± 2 °C, and 60% relative humidity. Sanger sequencing was performed to confirm mutant homozygosity and genotypes. Mutant genotypes were verified by Sanger sequencing. Transcript levels of anthocyanin biosynthetic structural genes (*CHS*, *F3H*, *DFR*, and *UF3GT*) were measured by RT-qPCR using *AtActin2* as the internal reference. Total anthocyanin content was determined spectrophotometrically at 530 nm, as described previously [32]. Total phenolic and total flavonoid contents were measured by HPLC using a Shimadzu LC-20A system (Shimadzu Corporation, Kyoto, Japan) equipped with a Shim Pack ODS C18 column (250 mm × 4.6 mm, 5 μm). The column temperature was maintained at 30 °C with a flow rate of 0.9 mL·min^−1^ and detection at 360 nm. The mobile phase consisted of acetonitrile (A) and 0.8% phosphoric acid aqueous solution (B). The gradient elution program was as follows: 0–15 min, 15% A; 15–30 min, 15–16% A; 30–35 min, 16–20% A; 35–40 min, 20–52% A; 40–45 min, 52–20% A.

### 2.9. Statistical Analysis

All experimental data are expressed as the mean ± standard deviation (SD) of three biological replicates. Two-way analysis of variance (ANOVA) combined with Duncan’s multiple range test (α = 0.05) was performed in SPSS 26.0 software. All visualization graphs were generated with GraphPad Prism 9.0.

## 3. Results and Analysis

### 3.1. Effects of Se Concentration on Antioxidant Substances in Mature Grains

**Figure 1:**
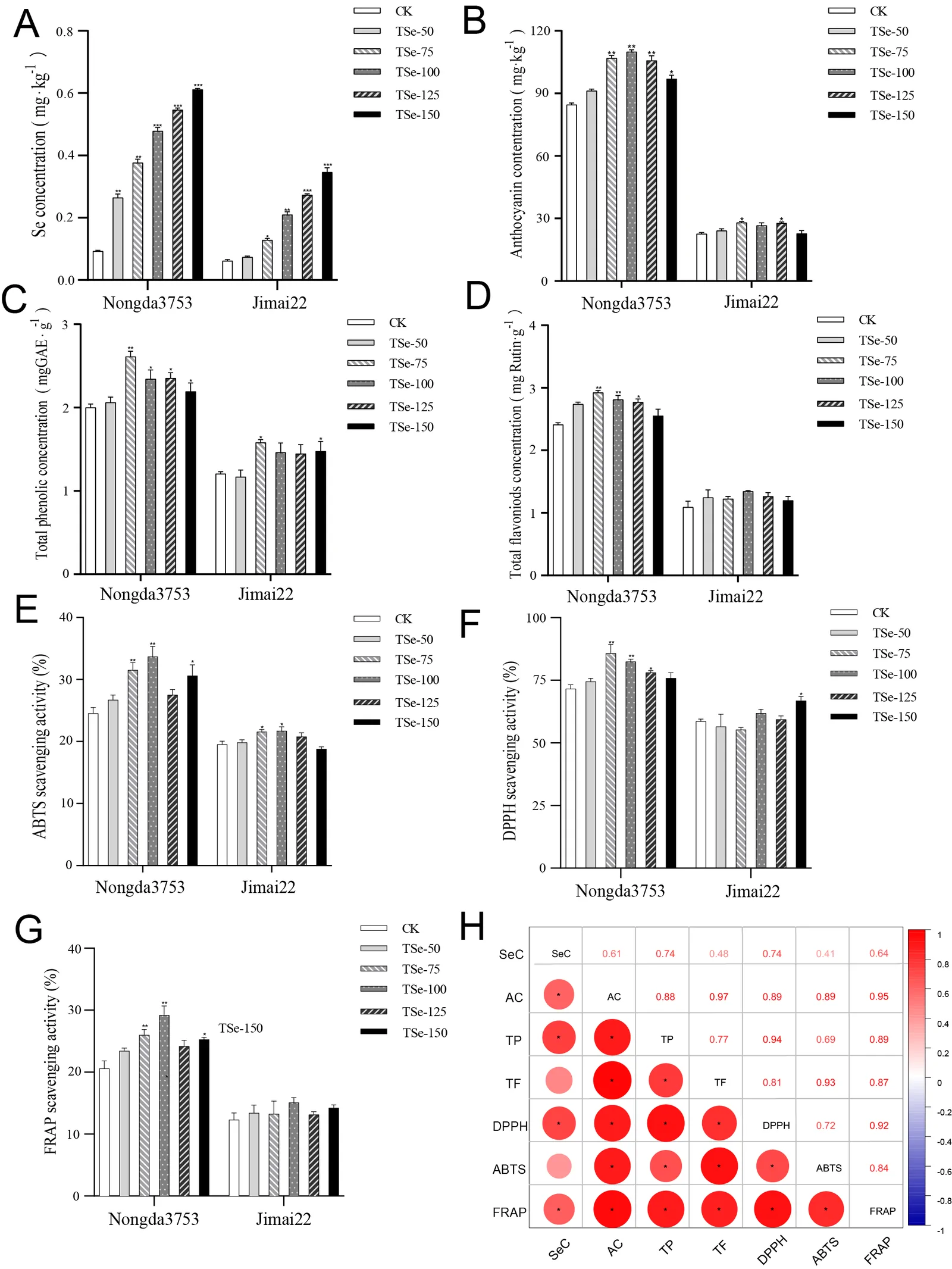
Effects of different Se fertilizer concentrations on antioxidant substances in mature grains of Nongda 3753 and Jimai 22. (**A**) Grain Se content. (**B**) Anthocyanin content. (**C**) Total phenolic content. (**D**) Total flavonoid content. (**E**) DPPH radical scavenging rate. (**F**) FRAP ferric reducing antioxidant power. (**G**) ABTS radical scavenging rate. (**H**) Pearson correlation heatmap among Se content, bioactive components, and antioxidant activities. CK, blank control; TSe-50 to TSe-150 represent sodium selenite treatments at 50–150 mg·L^−1^, respectively. *, **, and *** indicate significant differences from CK within the same cultivar at *p* < 0.05, *p* < 0.01, and *p* < 0.001, respectively. In the heatmap, SeC, Se content; AC, anthocyanin content; TP, total phenolic content; TF, total flavonoid content. Data are expressed as means ± SE (*n* = 3).

Grain Se content in both cultivars increased with elevated foliar Se dosage, reaching the maximum value under 150 mg·L^−1^ treatment (Figure 1A). For Nongda 3753, grain Se content reached 0.6079–0.6143 mg·kg^−1^, representing a 555.63% increase relative to the control, while grain Se content in Jimai 22 reached 0.3303–0.3547 mg·kg^−1^ (463.09% increase). Under identical Se application concentrations, Nongda 3753 exhibited significantly higher grain Se accumulation capacity than Jimai 22 (*p* < 0.01).

Anthocyanin, total phenolic, and total flavonoid contents, and DPPH, FRAP, and ABTS antioxidant activities all followed a unimodal rise-and-fall trend with increasing Se supply (Figure 1B–G). In Nongda 3753, total phenolics (2.55–2.68 mg GAE·g^−1^), total flavonoids (2.90–2.96 mg rutin·g^−1^), and DPPH scavenging rate (82.11–89.02%) peaked at 75 mg·L^−1^; anthocyanin content (108.75–110.58 mg·kg^−1^) and ABTS and FRAP activities reached their maxima at 100 mg·L^−1^ but also remained high and significantly above the control at 75 mg·L^−1^. Therefore, 75 mg·L^−1^ was selected as the optimal treatment for subsequent dynamic sampling and transcriptome analysis.

The Pearson correlation analysis demonstrated significant positive correlations between anthocyanin, total phenol, and total flavonoid and ABTS, DPPH, and FRAP activities (r = 0.69–0.97, *p* < 0.05). Grain Se concentration displayed only moderate positive correlations with antioxidant metabolite levels (r = 0.41–0.74) (Figure 1H).

### 3.2. Dynamics of Antioxidant Accumulation During Grain Filling Under Se Treatment

**Figure 2:**
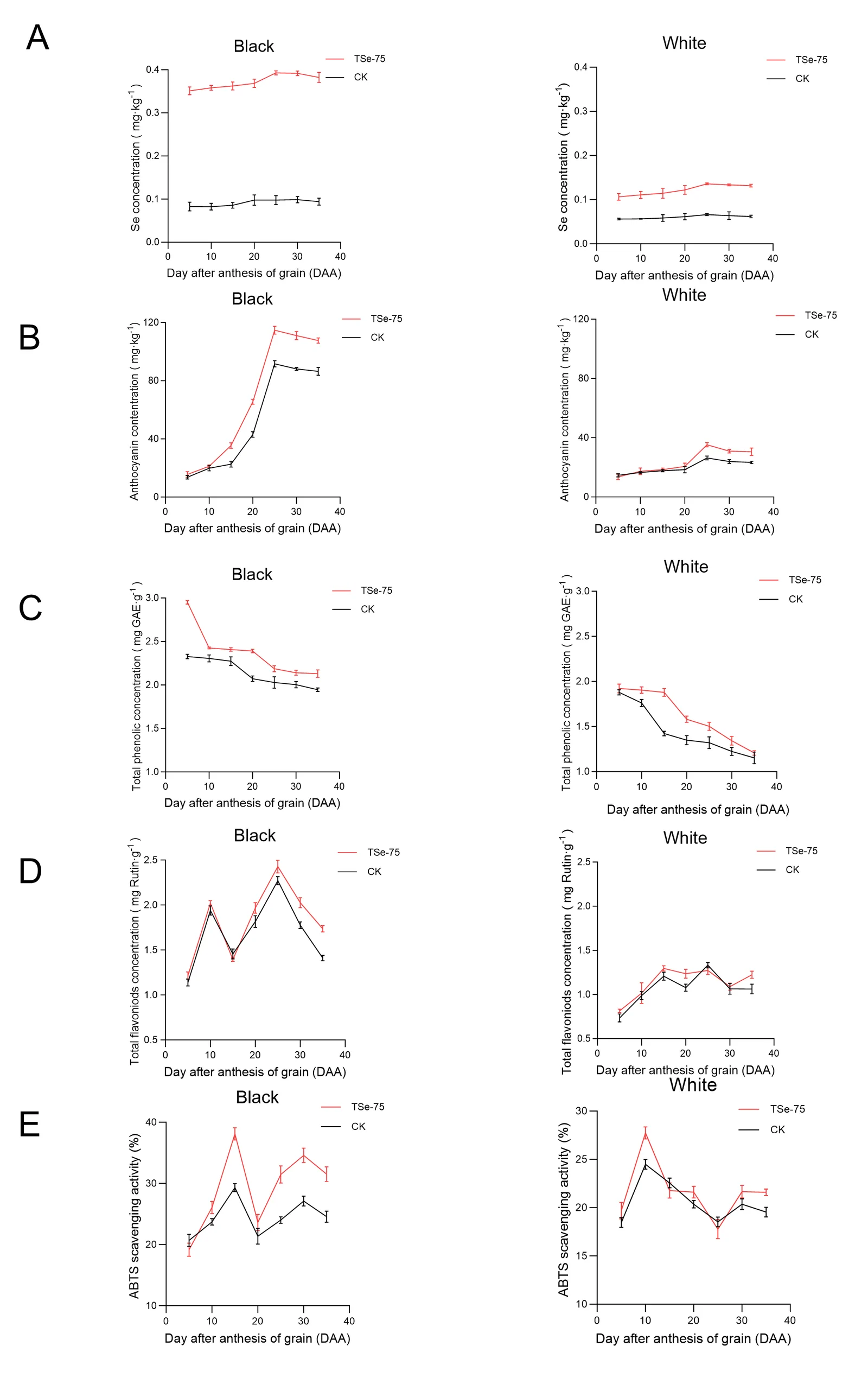
Dynamics of antioxidant substance accumulation in grains of Nongda 3753 and Jimai 22 during grain filling under 75 mg·L^−1^ Se treatment. (**A**) Grain Se content. (**B**) Anthocyanin content. (**C**) Total phenolic content. (**D**) Total flavonoid content. (**E**) ABTS radical scavenging rate. CK, control; SeT, 75 mg·L^−1^ sodium selenite treatment. Data are expressed as means ± SE (*n* = 3).

Under 75 mg·L^−1^ Se foliar spraying, continuous dynamic changes in grain Se and antioxidant metabolites were monitored across four grain-filling stages (Figure 2). Grain Se concentrations in both varieties gradually increased from 5 DAA, peaked at 25 DAA, and slightly declined before physiological maturity (Figure 2A). At 25 DAA, grain Se in Nongda 3753 reached 0.3898–0.3986 mg·kg^−1^, 301.77% higher than in the control group, and maintained a 305.90% increase at 35 DAA.

Grain anthocyanin accumulated rapidly from 10 to 25 DAA and achieved the peak value at 25 DAA (Figure 2B). Nongda 3753 accumulated 111.68–117.08 mg·kg^−1^ anthocyanin at this stage, 25.13% higher than CK. Total phenolic content reached the highest level at 5 DAA and gradually decreased throughout grain filling; Se treatment slowed the rate of decline in grain phenols (Figure 2C). Total flavonoid content presented a bimodal accumulation pattern with two peaks at 10 and 25 DAA, and the 25 DAA peak value was markedly higher (Figure 2D). ABTS radical scavenging capacity peaked at 15 DAA and exhibited a secondary minor peak at 25 DAA (Figure 2E).

### 3.3. Transcriptome Sequencing and Differential Expression Gene Analysis

An average of 45.12 million clean reads were obtained per sequencing library, with an average valid read ratio of 97.84%, an average Q30 value of 94.04%, and an average GC content of 55.99% (Table S2).

**Figure 3:**
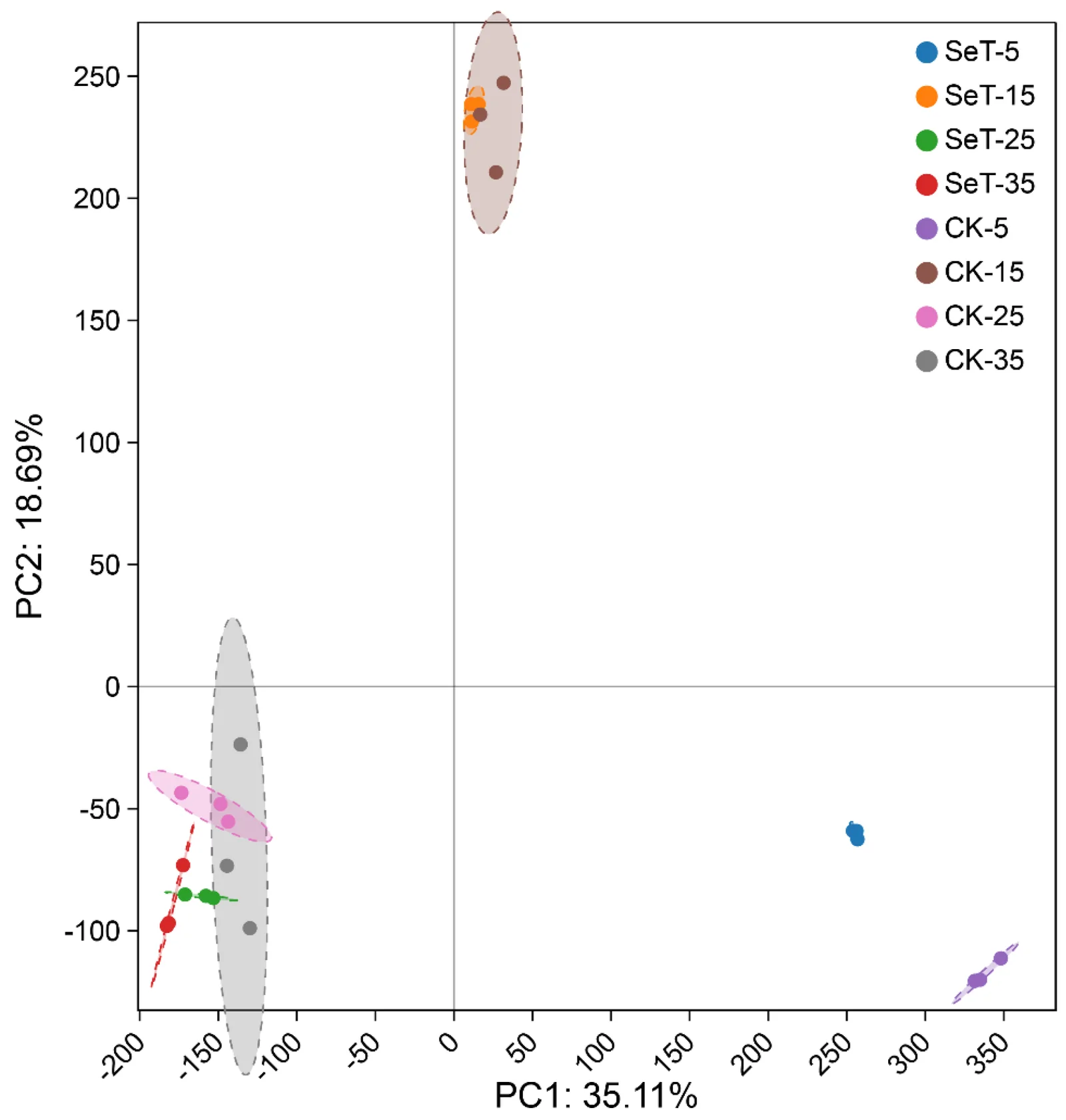
Principal component analysis (PCA) of transcriptomes from all samples. CK, control; SeT, 75 mg·L^−1^ sodium selenite treatment. The numbers 5, 15, 25, and 35 indicate days after anthesis (DAA). Values in parentheses for PC1 and PC2 represent the percentage of variance explained by each principal component.

Principal component analysis (PCA) revealed clear sample separation based on developmental stages: PC1 (35.11%) distinguished 5 DAA samples from 25/35 DAA materials, while PC2 (18.69%) separated 15 DAA samples from other stages. Within identical developmental time points, CK and SeT samples showed obvious transcriptional divergence, especially at 5 and 15 DAA (Figure 3).

**Figure 4:**
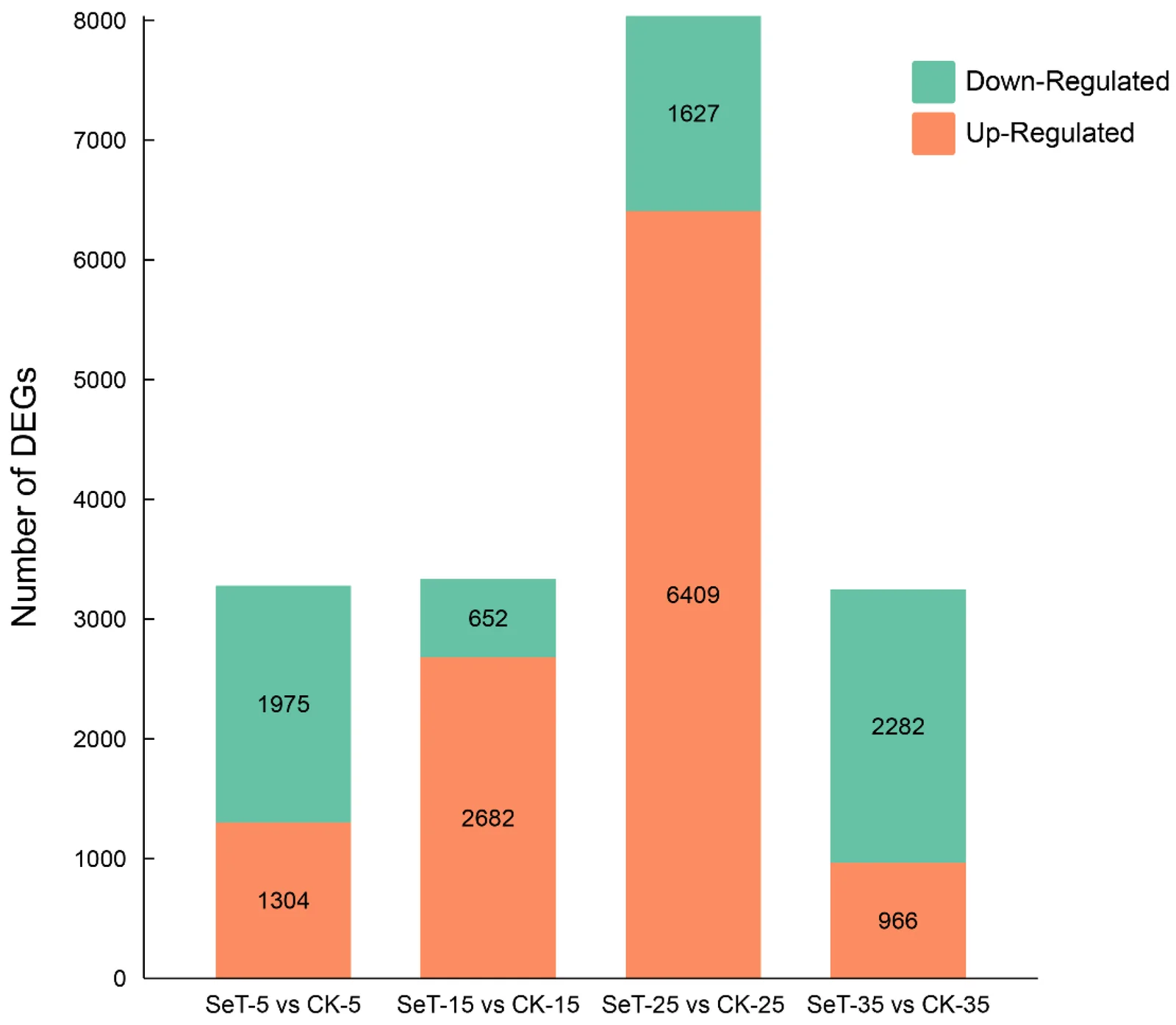
Statistics of differentially expressed genes (DEGs) at each developmental stage.

**Figure 5:**
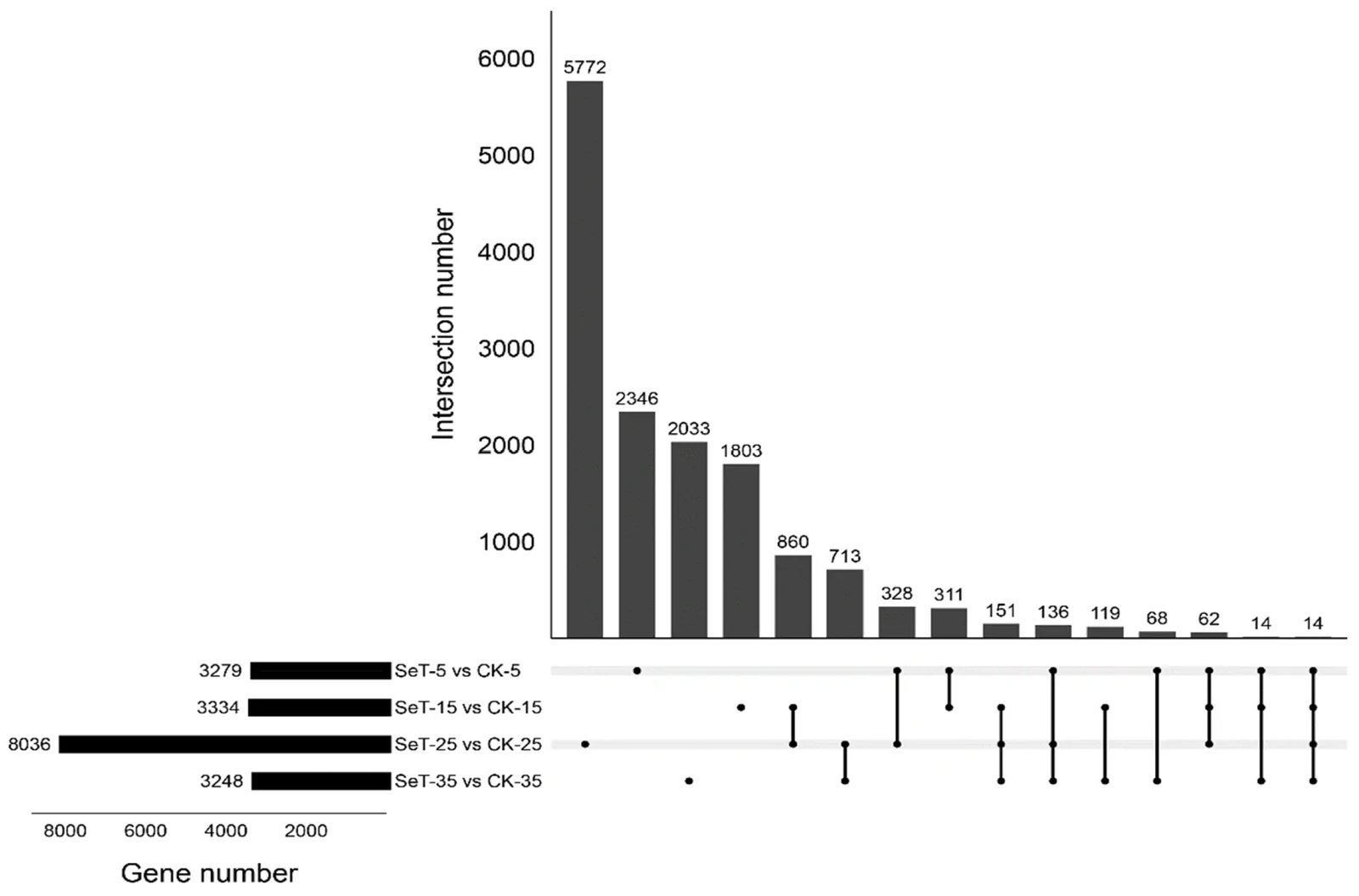
UpSet plot of DEGs across different developmental stages. The left bar chart shows the total number of DEGs at each stage. In the matrix, individual dots represent stage-specific DEGs, and connected dots represent DEGs shared among multiple stages.

The quantity of differentially expressed genes (DEGs) varied dramatically across grain-filling stages. The maximum number of DEGs (8036) was identified at 25 DAA, far exceeding the DEG counts at 15 DAA (3334), 5 DAA (3279) and 35 DAA (3248) (Figure 4). The up-/downregulation ratio exhibited stage-specific characteristics: at 5 DAA, downregulated DEGs dominated (1304 up vs. 1975 down); at 15 DAA, upregulated genes became dominant (2682 up vs. 652 down); at 25 DAA, the largest number of induced DEGs was observed (6409 up vs. 1627 down); at 35 DAA, repressed DEGs again constituted the majority (966 up vs. 2282 down). The UpSet plot analysis indicated that most DEGs were stage-specific, with only 11 shared DEGs detected across all four sampling points; the largest overlap of DEGs occurred between 15 and 25 DAA (876 shared genes) (Figure 5).

### 3.4. Go Enrichment Analysis

**Figure 6:**
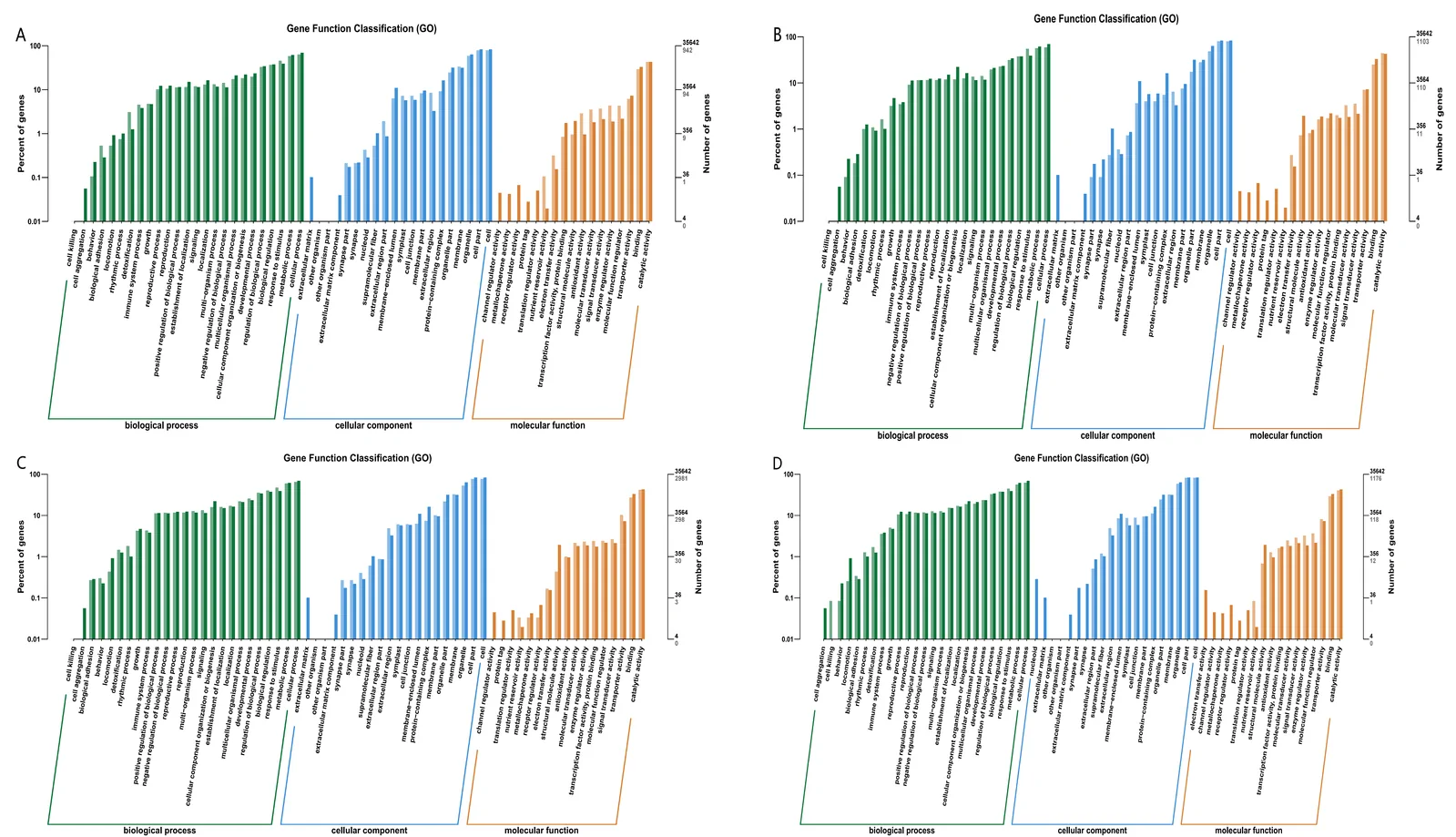
GO enrichment analysis of DEGs at different developmental stages. (**A**) 5 DAA. (**B**) 15 DAA. (**C**) 25 DAA. (**D**) 35 DAA. BP, biological process; CC, cellular component; MF, molecular function.

DEGs from distinct grain-filling stages displayed differences in enriched functional categories (Table S3, Figure 6). At 5 DAA, downregulated DEGs were significantly enriched in photosynthesis-related biological processes (protochlorophyllide reductase activity, chloroplast thylakoid membrane), while upregulated genes were mainly associated with proteolysis regulation and endopeptidase inhibitor activity (Figure 6A). At 15 DAA, induced DEGs were enriched in abiotic stress response pathways (drought, cold, ROS, ABA signaling), together with phenylpropanoid and secondary metabolite biosynthesis (Figure 6B). At 25 DAA, upregulated DEGs covered DNA replication, DNA unwinding, and MCM complex formation, as well as blue light and heat stress responses; repressed genes were enriched in organ senescence processes (Figure 6C). At 35 DAA, downregulated DEGs were enriched in negative regulation of ROS and oxidative cell death, while upregulated genes were enriched in genes encoding late flavonoid modification enzymes such as UDP-glucosyltransferases (Figure 6D).

### 3.5. KEGG Pathway Enrichment Analysis

**Figure 7:**
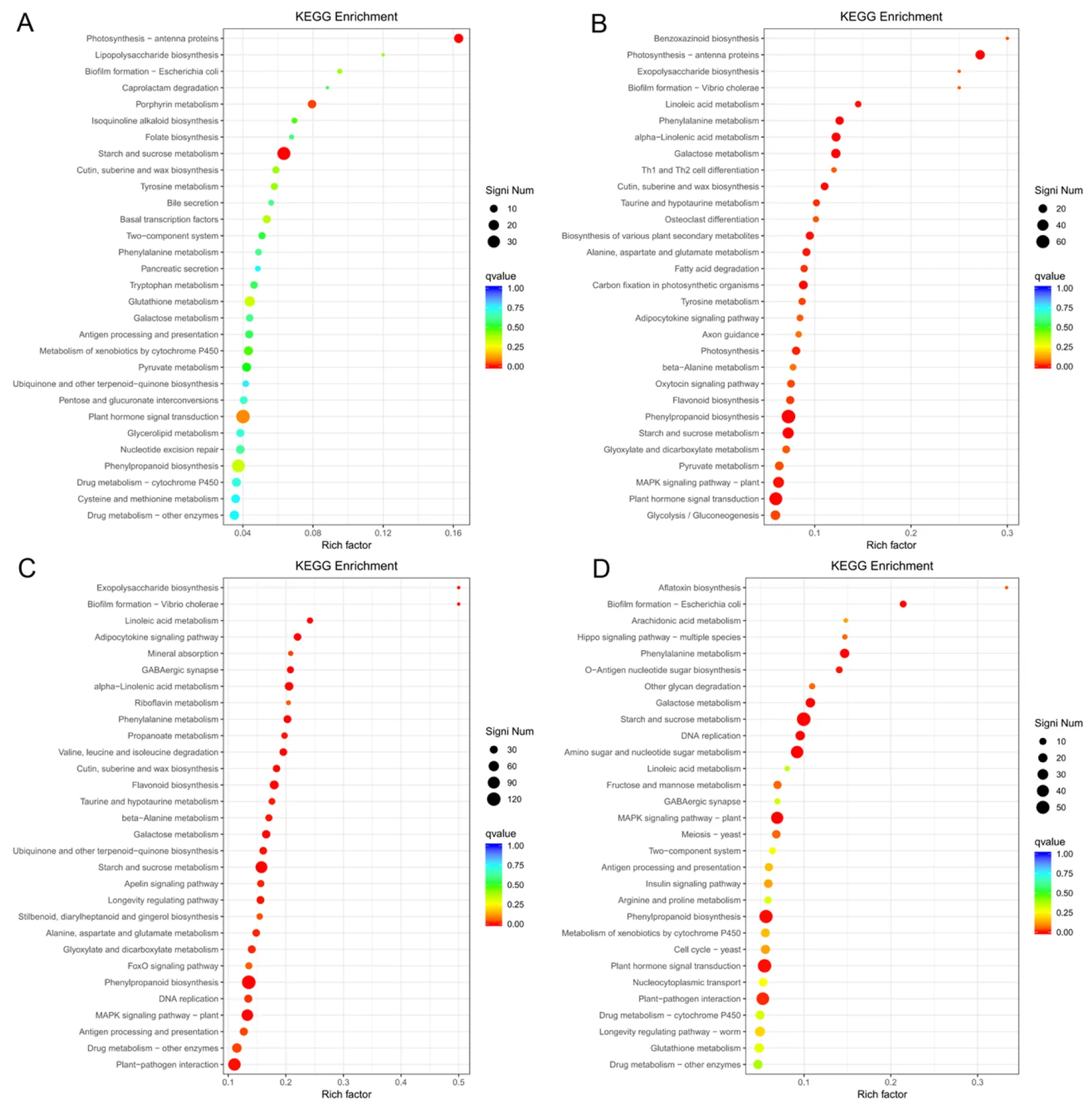
KEGG pathway enrichment analysis of DEGs at different developmental stages. (**A**) At 5 DAA. (**B**) At 15 DAA. (**C**) At 25 DAA. (**D**) At 35 DAA. Only the top 30 most significantly enriched pathways are shown. Dot size indicates the number of enriched genes, and color intensity reflects the level of significance (redder color indicates higher significance).

KEGG enrichment demonstrated obvious stage-specific metabolic reprogramming triggered by Se treatment (Figure 7). At 5 DAA, DEGs were enriched in photosynthetic antenna proteins, starch–sucrose metabolism, plant hormone signal transduction, phenylalanine and phenylpropanoid biosynthesis (Figure 7A). At 15 DAA, flavonoid biosynthesis, linoleic acid metabolism, and MAPK signaling pathways were additionally significantly enriched (Figure 7B). At 25 DAA, the broadest spectrum of pathways was activated, including carbon/nitrogen metabolism, mineral absorption, plant–pathogen interaction, DNA replication, cutin/wax synthesis, and core secondary metabolic routes (phenylpropanoid and flavonoid biosynthesis); uniquely, the anthocyanin biosynthesis pathway was significantly enriched only at 25 DAA (Figure 7C). At 35 DAA, pathway enrichment contracted to basal carbon metabolism, hormone signaling, and DNA replication (Figure 7D).

**Figure 8:**
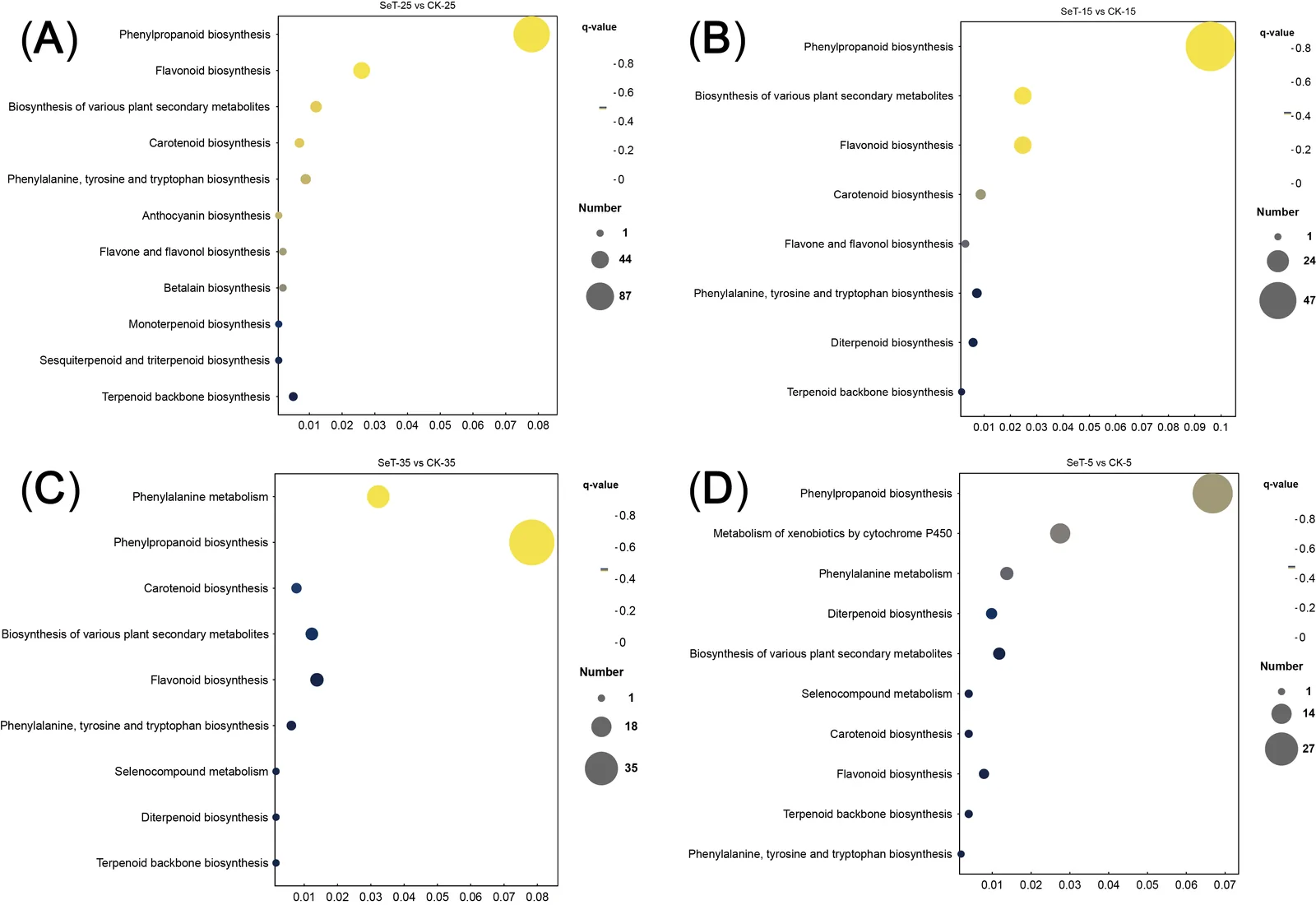
KEGG enrichment analysis of secondary metabolism-related pathways of DEGs at different grain-filling stages. (**A**) At 5 DAA. (**B**) At 15 DAA. (**C**) At 25 DAA. (**D**) At 35 DAA. Dot size indicates the number of enriched genes, and color represents the q-value (yellower color indicates higher significance).

Targeted enrichment analysis of secondary metabolic pathways further verified temporal activation patterns (Figure 8). In early 5 DAA samples, upstream precursor synthesis pathways were mainly enriched; at 15 DAA flavonoid and carotenoid biosynthesis was initiated; 25 DAA represented the peak activation stage of the complete anthocyanin pathway; during late filling (35 DAA), enrichment returned to basal phenylalanine metabolic processes.

### 3.6. Identification and Expression Profiles of Anthocyanin Biosynthesis-Related Genes

**Figure 9:**
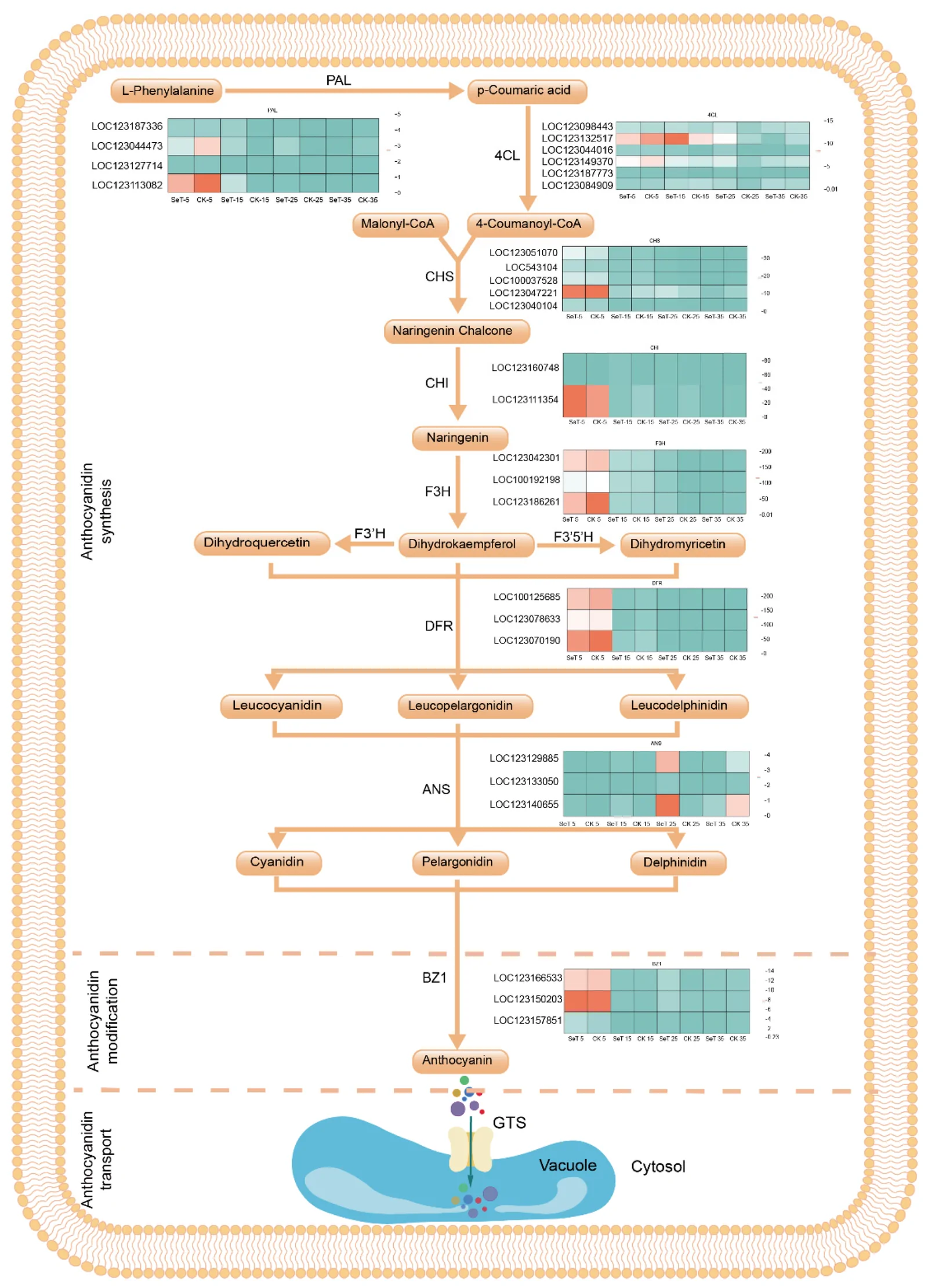
Metabolic pathway of anthocyanin biosynthesis regulated by Se and expression heatmap of key genes in black wheat grains. Heatmap colors represent relative gene expression levels (TPM values normalized by Z-score), with redder color indicating higher expression. PAL, phenylalanine ammonia-lyase; 4CL, 4-coumarate-CoA ligase; CHS, chalcone synthase; CHI, chalcone isomerase; F3H, flavanone 3-hydroxylase; F3′H, flavonoid 3′-hydroxylase; F3′5′H, flavonoid 3′,5′-hydroxylase; DFR, dihydroflavonol 4-reductase; ANS, anthocyanidin synthase; BZ1, anthocyanidin 3-*O*-glucosyltransferase; GST, glutathione S-transferase. CK-5 to CK-35 denote control samples at 5–35 DAA, and SeT-5 to SeT-35 denote the corresponding Se-treated samples.

In total, 29 DEGs involved in the full anthocyanin biosynthetic cascade were screened (Figure 9), covering the complete process from upstream precursor synthesis to downstream modification, including upstream genes *PAL* (4) and *4CL* (6), flavonoid backbone biosynthesis genes *CHS* (5), *CHI* (2), and *F3H* (3), downstream genes *DFR* (3) and *ANS* (3), and the modification gene *BZ1* (3) (Figure 9). At 5 DAA, the expression levels of upstream and midstream structural genes showed no significant differences between the CK and SeT groups. At 15 DAA, partial 4CL transcripts were induced by Se. At 25 DAA, most upstream flavonoid genes declined with grain development, whereas the terminal rate-limiting gene ANS was specifically upregulated under Se treatment, consistent with the peak anthocyanin accumulation phenotype at this stage. Two ANS homologous genes (LOC123140655, LOC123129885) exhibited markedly higher TPM values in the SeT-25 samples, accompanied by synchronous upregulation of the glycosylation gene BZ1.

### 3.7. Screening of Anthocyanin-Regulated Transcription Factors

**Figure 10:**
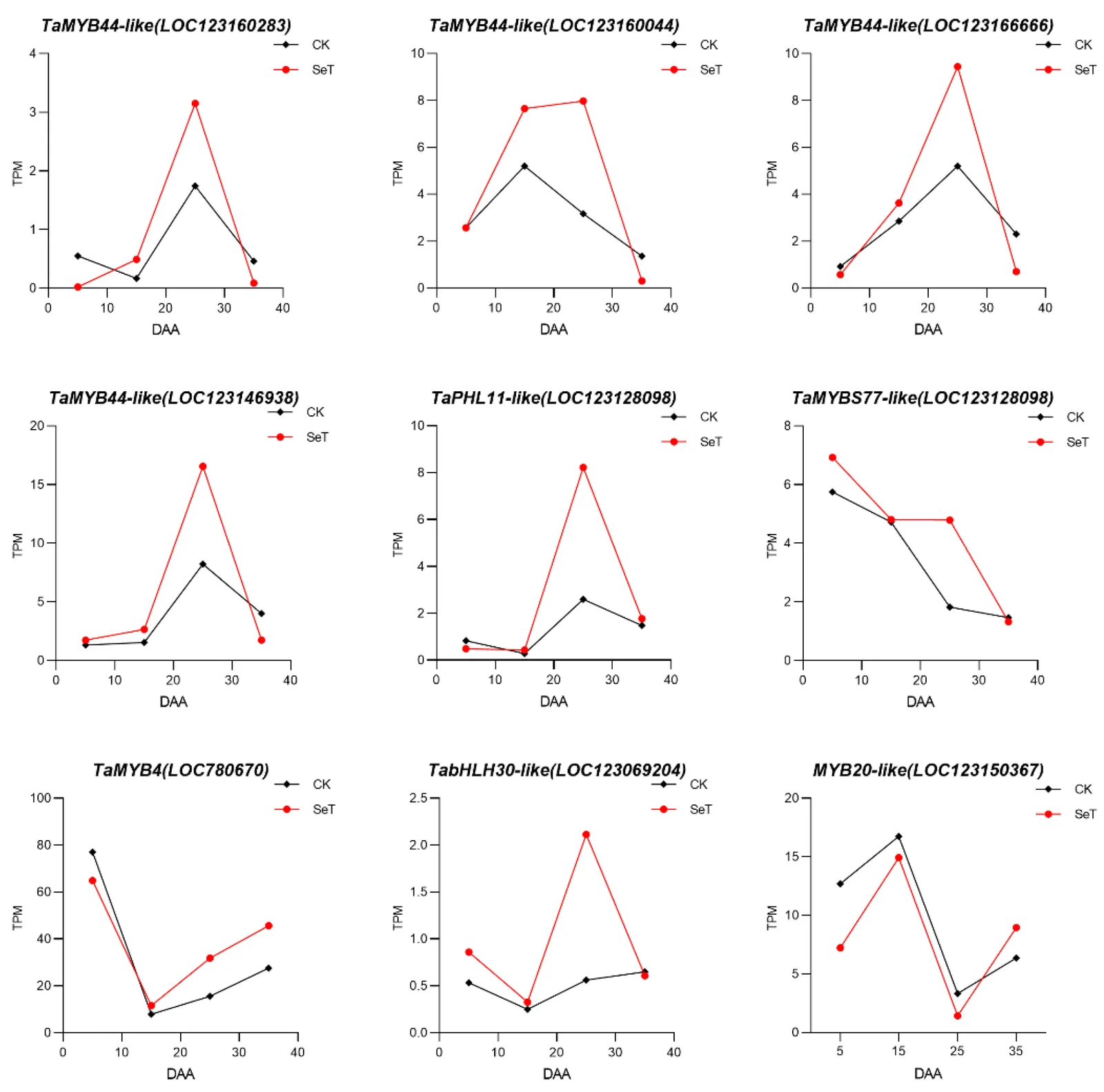
Expression patterns of candidate transcription factors related to anthocyanin biosynthesis during grain filling. Heatmap colors represent relative gene expression levels (TPM values normalized by Z-score). CK-5 to CK-35 denote control samples at 5–35 DAA, and SeT-5 to SeT-35 denote the corresponding Se-treated samples.

From 159 Se-responsive transcription factors (111 MYB, 48 bHLH), nine candidate TFs were identified based on their expression dynamics and temporal correlation with anthocyanin accumulation (Figure 10). Four *TaMYB44-like* homologs (*LOC123160283*, *LOC123160044*, *LOC123146938*, and *LOC123180432*) and one *TaPHL11-like* gene (*LOC123042190*) maintained low expression at early filling (5–15 DAA) and peaked at 25 DAA, with significantly higher expression under Se treatment. The candidate repressor *TaMYB20-like* (*LOC123150367*) showed maximal transcript abundance at 5 DAA, continuously decreased to a minimum at 25 DAA and slightly recovered at 35 DAA; its expression level was consistently lower in Se-treated grains across all stages. *TaMYB4 (LOC123058319)* exhibited high expression at 5 DAA (CK > SeT), declined at 15 DAA, and gradually increased from 25 to 35 DAA, with Se-induced upregulation. TabHLH30-like expression continuously increased from 15 DAA and reached peak abundance at 25 DAA under Se supply. *TaMYBS77-like* (*LOC123111228*) showed an overall declining trend during grain filling, with the highest expression at 5 DAA (SeT > CK). Among the bHLH family, *TabHLH30-like* (*LOC123166503*) displayed low expression at 5 DAA, increased from 15 DAA onward, and peaked at 25 DAA, with higher expression in SeT than in CK.

### 3.8. RT-qPCR Validation of Transcriptome Data

**Figure 11:**
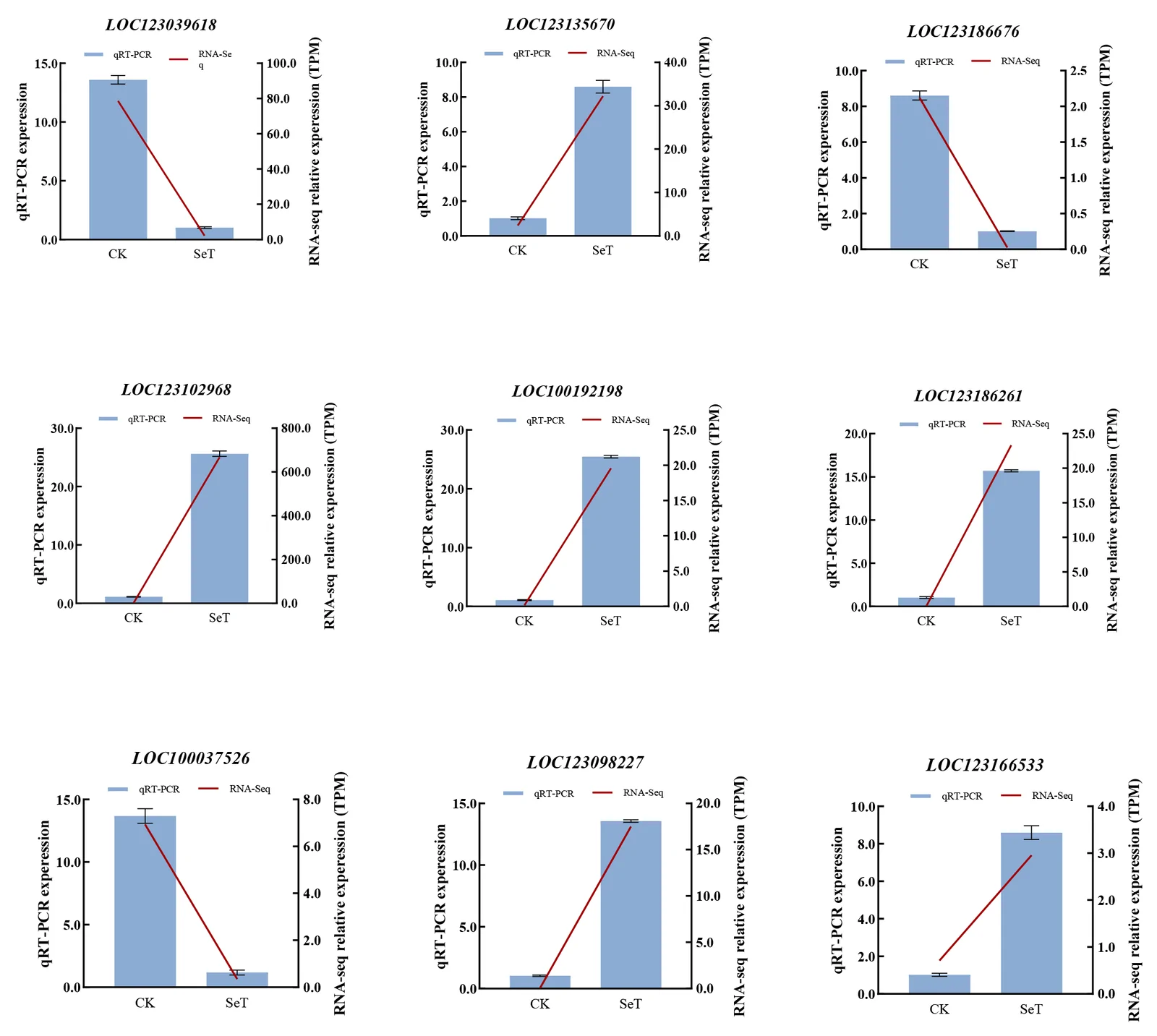
Validation of differentially expressed genes by RT-qPCR. The blue bars represent the RNA-seq data (TPM values, left *Y*-axis), and the red lines represent the RT-qPCR data (relative expression, right *Y*-axis). Data are expressed as means ± SD (n = 3).

Nine randomly selected DEGs were subjected to RT-qPCR quantification. The relative expression trends detected by real-time PCR were highly consistent with the TPM expression profiles obtained from RNA-seq, confirming the reliability of the transcriptome differential expression screening results (Figure 11).

### 3.9. Expression Characteristics and Bioinformatics Analysis of Tamyb20-like

**Figure 12:**
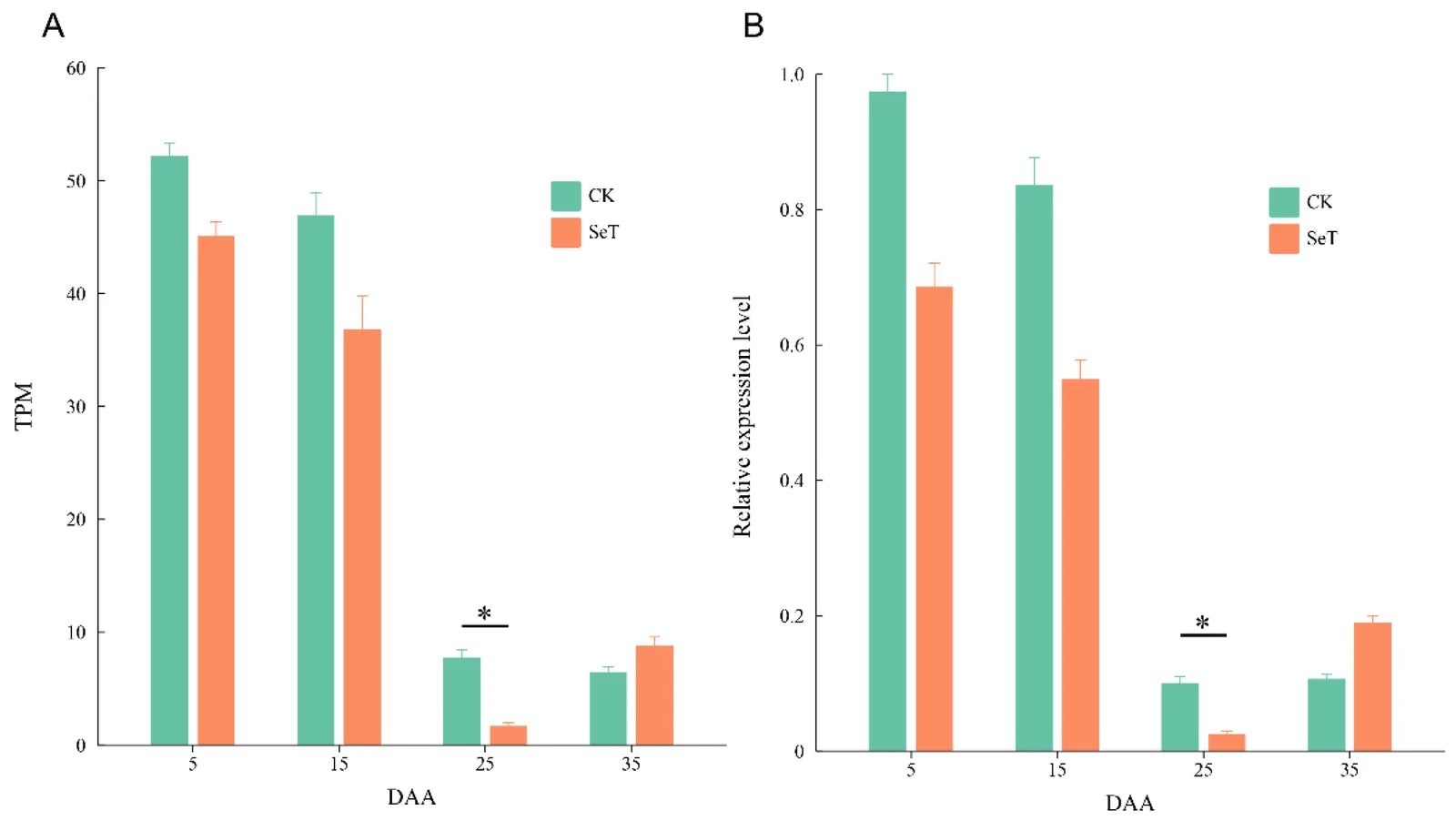
Expression pattern of TaMYB20-like during grain filling. (**A**) Transcriptome sequencing expression levels (TPM). (**B**) Relative expression levels determined by qRT-PCR. * indicates a significant difference between SeT and CK at the same stage (*p* < 0.05). CK, control; SeT, 75 mg·L^−1^ sodium selenite treatment. Data are expressed as means ± SD (n = 3).

Transcriptome data showed that *TaMYB20-like* was most highly expressed at 5 DAA, progressively declined during grain filling, reached its lowest level at 25 DAA, and slightly recovered at 35 DAA, with consistently lower expression in SeT than in CK across all stages (Figure 12A). RT-qPCR validation results were consistent with the RNA-seq data (Figure 12B). The expression pattern of this gene was opposite to that of anthocyanin accumulation.

**Figure 13:**
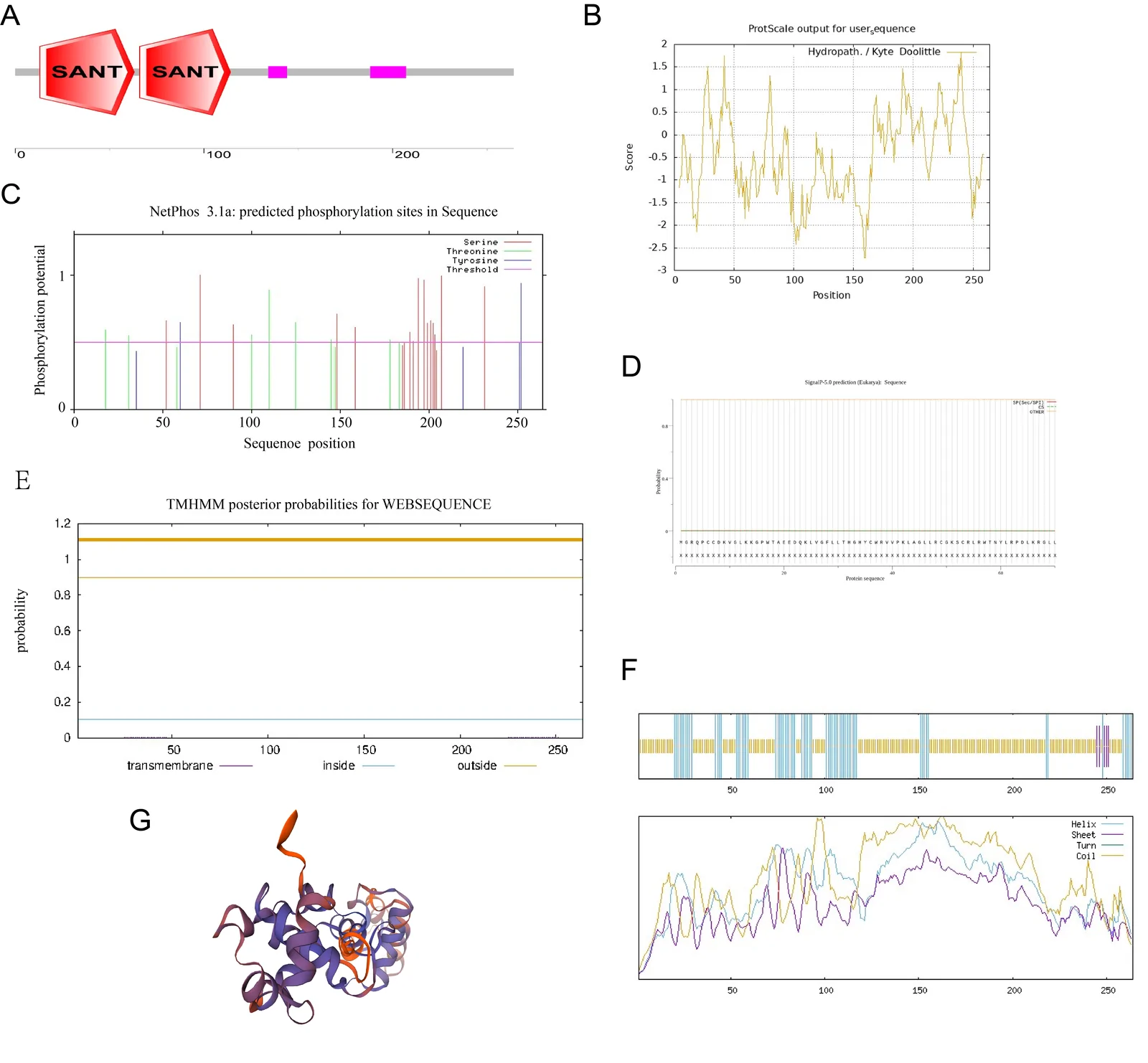
Bioinformatics analysis of TaMYB20-like protein. (**A**) Conserved domain prediction. (**B**) Hydrophilicity/hydrophobicity analysis. (**C**) Phosphorylation site prediction. (**D**) Signal peptide prediction. (**E**) Transmembrane domain prediction. (**F**) Secondary structure prediction. (**G**) Tertiary structure homology modeling.

**Figure 14:**
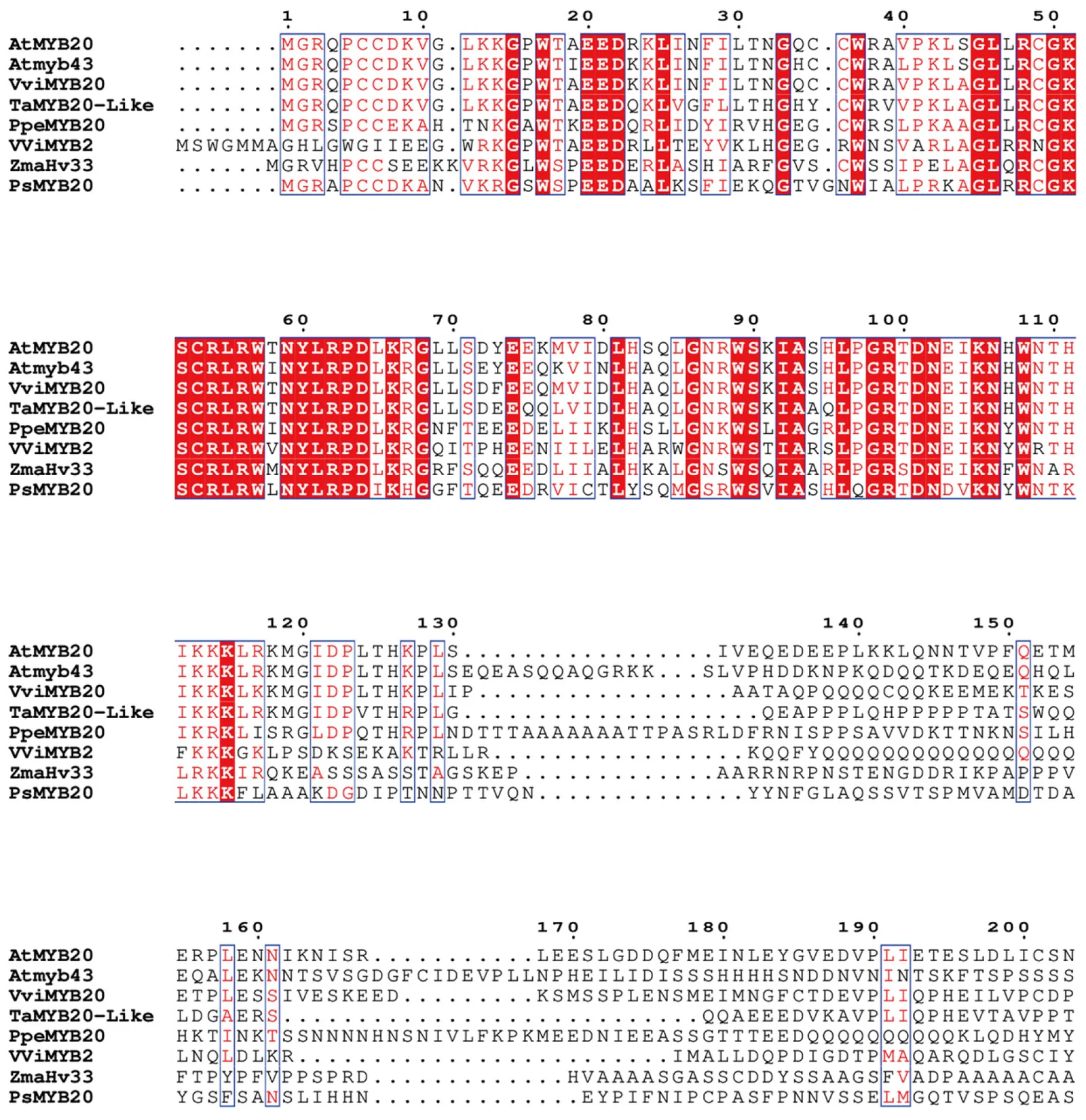
Multiple sequence alignment of TaMYB20-like and its homologous proteins. *At*, *Arabidopsis thaliana*; *Ppe*, *Prunus persica*; *Vvi*, *Vitis vinifera*; *Zma*, *Zea mays*; *Ps*, *Paeonia suffruticosa*; *Ta*, *Triticum aestivum* L.

**Figure 15:**
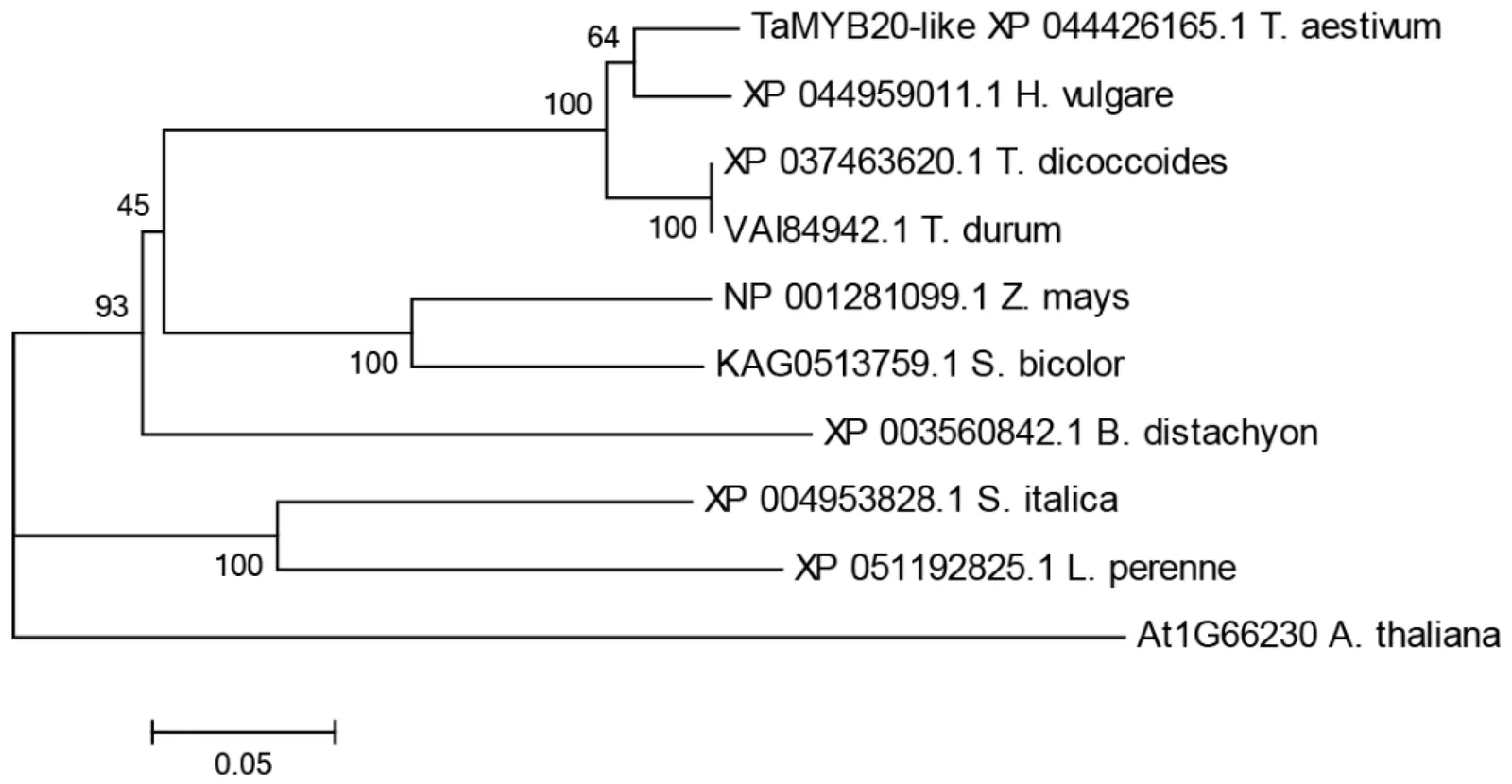
Phylogenetic tree of TaMYB20-like and representative MYB homologous proteins from grasses and Arabidopsis thaliana. The tree was constructed using the neighbor-joining (NJ) method with 1000 bootstrap replicates. Bootstrap support values (≥50%) are shown on the branches. The red arrow indicates the TaMYB20-like protein.

*TaMYB20-like* protein contained two SANT domains (Figure 13A), with a molecular weight of 28.92 kDa, an isoelectric point of 6.13, a grand average of hydropathicity (GRAVY) of −0.494 (Figure 13B), an instability index of 59.12, and an aliphatic index of 73.90. The protein harbored 24 phosphorylation sites (15 Ser, 7 Thr, and 2 Tyr residues) (Figure 13C) and lacked signal peptides (Figure 13D) and transmembrane domains (Figure 13E). The secondary structure comprised 25.76% α-helix, 2.27% extended strand, and 71.97% random coil, and homology modeling of the tertiary structure further confirmed a predominance of α-helix and random coil (Figure 13F,G). Multiple sequence alignment revealed high interspecies conservation, and phylogenetic tree analysis confirmed close evolutionary homology with Arabidopsis AtMYB20 (Figure 14 and Figure 15).

### 3.10. Functional Validation via Arabidopsis AtMYB20 Mutant

**Figure 16:**
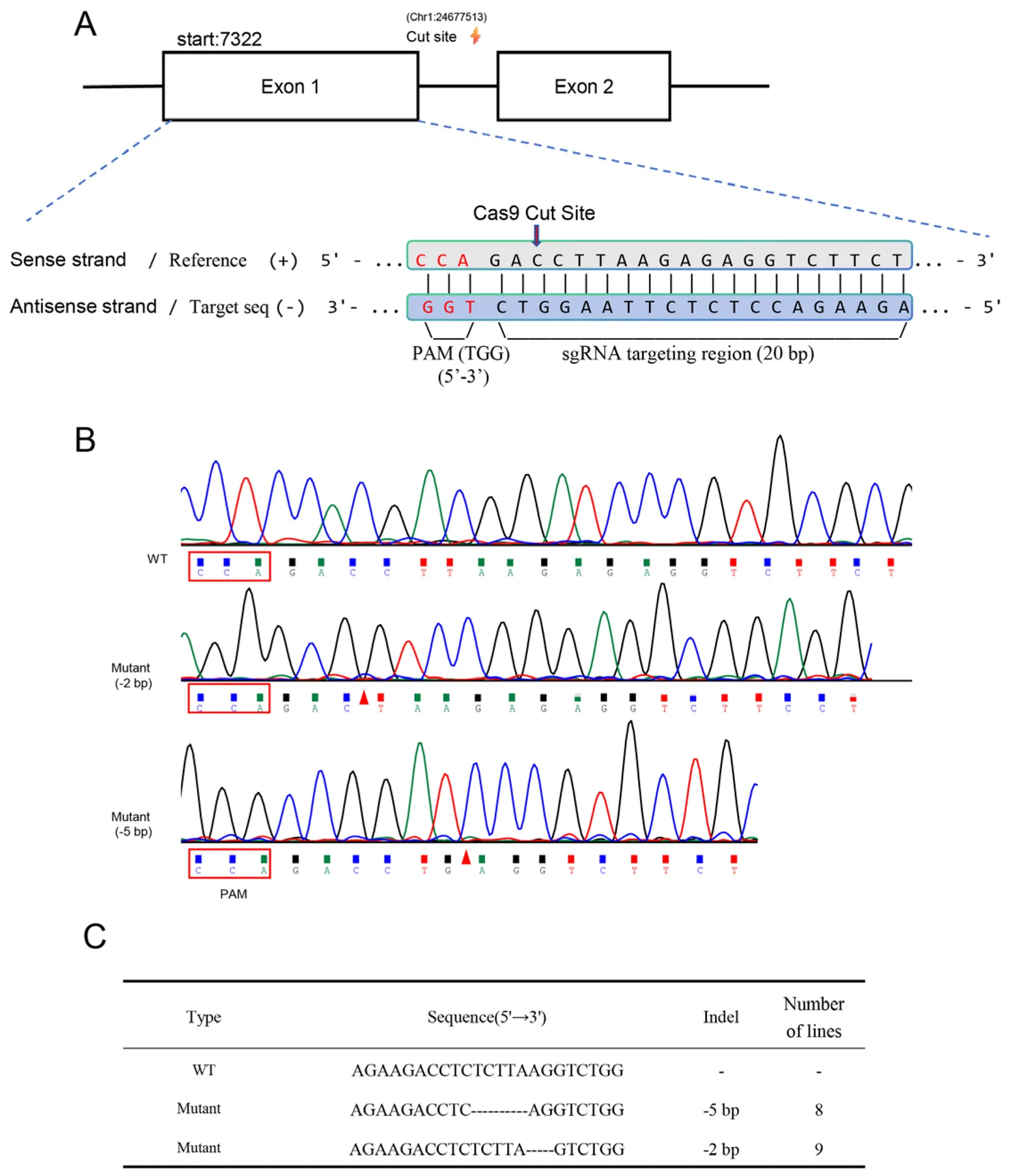
Identification of CRISPR/Cas9-mediated AtMYB20 mutants. (**A**) Schematic diagram of the AtMYB20 gene structure and the double-stranded sequence of the target region, with the red arrow indicating the Cas9 cleavage site. (**B**) Representative Sanger sequencing chromatograms of the target region in wild-type (WT) and two homozygous mutant lines (−2 bp and −5 bp); the red box on the left highlights the PAM sequence, and the red triangles indicate the positions of the base deletions. (**C**) Sequence alignment and segregation statistics of T2 mutants, with dashes (-) representing the deleted bases.

**Figure 17:**
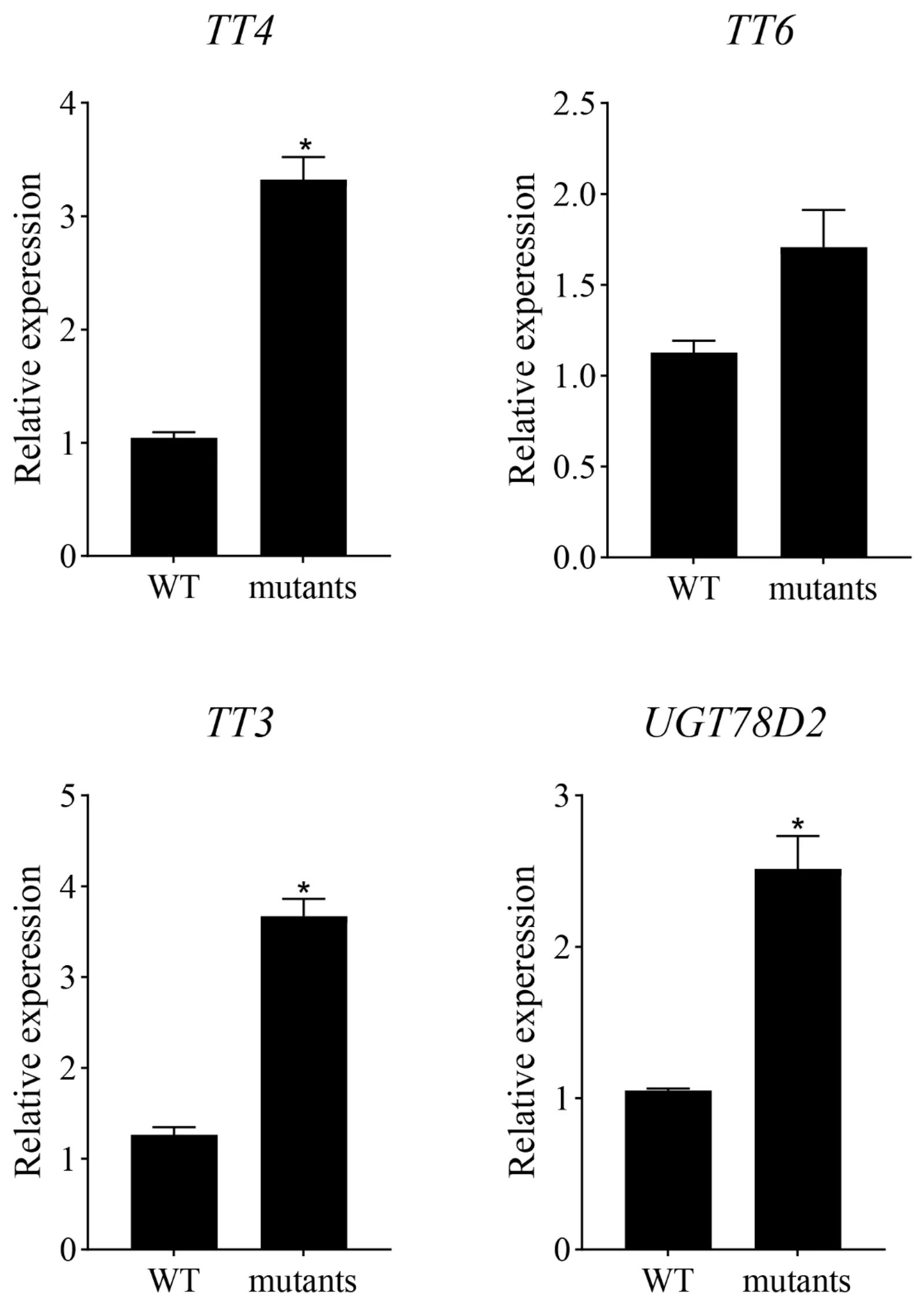
Expression analysis of key anthocyanin biosynthesis genes in the AtMYB20 mutant. Relative expression levels were determined using the wild type (WT) as the control (set to 1.0). *TT4*, chalcone synthase gene; *TT6*, flavanone 3-hydroxylase gene; *TT3*, dihydroflavonol 4-reductase gene; *UGT78D2*, UDP-glucose: flavonoid 3-*O*-glucosyltransferase gene. * indicates a significant difference compared with WT (*p* < 0.05). Data are expressed as means ± SD (n = 3).

**Figure 18:**
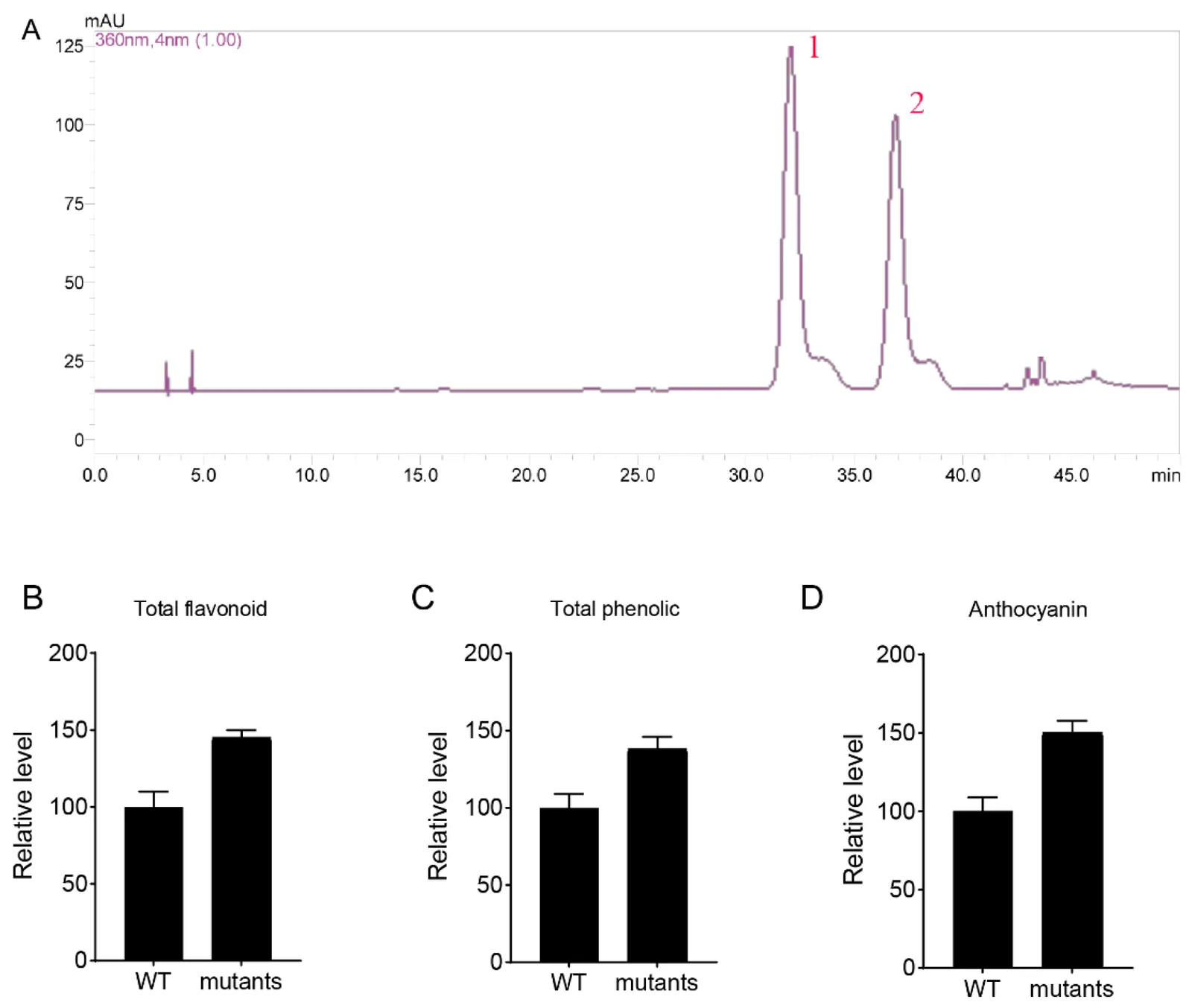
Analysis of antioxidant substance contents in AtMYB20 mutant seedlings. (**A**) HPLC chromatogram of mixed standards (peak 1, rutin; peak 2, gallic acid). (**B**) Relative total flavonoid content in the mutant. (**C**) Relative total phenolic content in the mutant. (**D**) Relative anthocyanin content in the mutant. Wild-type (WT) values were set as 100%. Data are expressed as means ± SD (n = 3).

Sanger sequencing verified two types of homozygous AtMYB20 CRISPR mutant lines (5 bp deletion and 2 bp deletion) (Figure 16). Compared with Col-0 wild-type seedlings, the key anthocyanin structural genes *TT4*, *TT3*, and *UGT78D2* were significantly upregulated in mutant plants (*p* < 0.05), while *TT6* displayed an upward trend without statistical significance (Figure 17). Metabolite quantification results showed that the total flavonoid, total phenolic, and anthocyanin contents of mutant seedlings reached 145%, 138%, and 153% of wild-type levels, respectively (Figure 18).

## 4. Discussion

### 4.1. Optimization of Selenium Fertilizer Concentration and Varietal Differences

The current study identified 75 mg·L^−1^ sodium selenite as the optimal foliar application dosage for black wheat Nongda 3753. Exogenous Se exhibits a typical hormetic effect on grain antioxidant biosynthesis: low concentrations promote pigment accumulation, while excessive Se causes metabolic inhibition, consistent with prior reports on pigmented cereals [15,16]. High Se concentrations induce phytotoxicity and disrupt secondary metabolic flux, which explains the descending trend in anthocyanin levels above 75–100 mg·L^−1^ [33]. Black wheat Nongda 3753 possessed superior Se absorption and antioxidant accumulation capacity relative to white wheat Jimai 22, which originates from inherent genetic differences in phenylpropanoid pathway gene expression between pigmented and non-pigmented wheat varieties [5,34].

Consistent with previous reports on pigmented cereal genotypes, black-grained wheat showed higher grain Se accumulation capacity than white-grained Jimai 22 under identical foliar Se supply conditions. This genotypic difference is partly attributed to the distinct tissue characteristics of the grain pericarp and aleurone layers. Black wheat carries active anthocyanin- and phenylpropanoid-related transcriptional programs in these outer grain tissues, which are transcriptionally quiescent in ordinary white wheat. Elevated basal activity of the phenylpropanoid pathway may modify tissue chelating properties and secondary metabolite pools, indirectly facilitating Se assimilation and deposition within grains. Furthermore, differences in mineral translocation efficiency from source leaves to sink grains between the two genotypes may also contribute to the observed divergence in final grain Se concentrations. Nevertheless, the precise molecular mechanisms governing genotypic variation in grain Se enrichment remain incompletely resolved; further comparative transcriptomic and ion transport studies are required to dissect the core genes controlling differential Se accumulation in pigmented versus non-pigmented wheat germplasm.

The correlation analysis showed that grain antioxidant capacity mainly depends on phenol and flavonoid concentrations rather than Se content itself, matching consensus conclusions on cereal antioxidant systems [35,36].

Another important limitation of this work originates from our field experimental setup: all field trials were performed at one experimental station within a single wheat growing season. Agronomic factors, including seasonal rainfall, temperature fluctuations, and light conditions, can substantially modulate grain selenium absorption and the biosynthesis of phenolic and flavonoid metabolites. Accordingly, the optimal foliar selenium concentration screened in the present study may be affected by year-to-year or location-specific environmental differences. Multisite and multi-season field trials will be required in future research to validate the generalizability of our recommended selenium application dosage under diverse agro-ecological conditions.

It should be noted that the grain selenium concentrations reported in Figure 1 reflect tissue-level Se status (mg·kg^−1^ dry weight), rather than the total fraction of applied selenium recovered within plant tissues. In the present field experiment, we did not perform isotopic tracing or whole-plant Se mass-balance sampling, so the exact percentage of sprayed Se taken up by wheat plants cannot be directly calculated. Isotope-labelled foliar Se experiments under controlled pot conditions have demonstrated that 43–56% of applied selenium can be recovered in wheat above-ground biomass; nevertheless, such high recovery values are difficult to achieve under practical field conditions owing to spray droplet loss and rainfall-mediated wash-off. After leaf absorption, a substantial proportion of selenium can be remobilized from vegetative tissues and translocated into developing grains [37].

### 4.2. Temporal Accumulation of Antioxidants During Grain Filling

In this study, the grain filling period was divided into four critical stages to characterize the spatiotemporal accumulation patterns of antioxidants. Anthocyanins reached their maximum at 25 DAA, consistent with previous observations in purple wheat [22]. Total phenolics reached their maximum at 5 DAA and subsequently declined continuously, which may be attributed to the “dilution effect” associated with dry matter accumulation and phenolic degradation. However, selenium treatment mitigated the rate of decline, likely by maintaining biosynthetic capacity through the activation of key enzymes in phenolic metabolic pathways [15]. Total flavonoids exhibited a bimodal pattern, reflecting the dynamic equilibrium between flavonoid synthesis and transformation at different developmental stages. The bimodal pattern of antioxidant activity was closely correlated with the accumulation of secondary metabolites: the peak at 15 DAA likely originated from early phenolic contributions, while the resurgence at 30 DAA was associated with the substantial accumulation of anthocyanins.

### 4.3. Transcriptional Network of Selenium-Regulated Anthocyanin Biosynthesis

Transcriptome data confirmed 25 DAA as the core stage of Se-mediated secondary metabolic reprogramming, when the complete anthocyanin biosynthetic pathway was uniquely enriched among all developmental time points. The terminal structural gene ANS was specifically induced by Se supply, supporting its function as the rate-limiting enzyme in anthocyanin monomer synthesis [38,39]. A series of positive MYB and bHLH transcription factors were identified, which coordinate the activation of upstream phenylpropanoid and downstream flavonoid genes during mid-to-late grain filling. The stage-specific transcriptional response to Se originates from developmental shifts in grain physiological status and temporal activation of MBW regulatory complexes.

Combining physiological phenotypes and transcriptomic reprogramming data, we can explain why the anthocyanin biosynthesis pathway is significantly enriched specifically at 25 DAA. Physiologically, anthocyanin content reaches its maximum value at 25 DAA under foliar selenium treatment. Transcriptomic evidence demonstrates that multiple structural genes responsible for anthocyanin biosynthesis exhibit a sharp transcriptional burst specifically at 25 DAA, matching the phenotypic peak of pigment accumulation. The 25 DAA stage constitutes an intrinsic critical developmental window for anthocyanin accumulation in black wheat grains. Exogenous selenium could synchronize and amplify this development-dependent transcriptional activation of anthocyanin-related genes. Before 25 DAA, the full transcriptional machinery for anthocyanin biosynthesis is not completely activated during grain development. After 25 DAA, gradual physiological maturation of grains suppresses the expression of secondary-metabolism-related genes. Collectively, these developmental characteristics lead to the significant enrichment specifically of the anthocyanin pathway at 25 DAA.

### 4.4. Negative Regulatory Function of Tamyb20-like

The TaMYB20-like expression pattern was negatively correlated with grain anthocyanin abundance, and its consistent downregulation under Se treatment implied its role as a transcriptional repressor. Functional testing using Arabidopsis AtMYB20 mutants indicated that loss of AtMYB20 function releases inhibition on flavonoid pathway structural genes, including CHS, DFR, and UF3GT, leading to elevated levels of anthocyanins, total phenolics, and total flavonoids. Previous research demonstrated that AtMYB20 participates in the repression of phenylalanine and lignin metabolism [40]. We propose that under normal growth conditions, TaMYB20-like directs carbon flux toward lignin biosynthesis and restricts flavonoid production; exogenous Se suppresses TaMYB20-like transcription, thereby relieving metabolic inhibition and facilitating anthocyanin accumulation in black wheat grains.

It is necessary to explicitly address several inherent limitations of the present study. First, the functional inference of wheat TaMYB20-like is mainly supported by heterologous phenotypic data obtained from Arabidopsis AtMYB20 CRISPR mutant lines. Heterologous assay results in model plants cannot fully reflect the authentic biological function of endogenous TaMYB20-like in wheat. Stable genetic transformation techniques, including VIGS, gene overexpression, or gene editing in wheat, are required in future research to directly validate its in vivo function in anthocyanin repression.

Second, the molecular regulatory cascade linking the selenium signal to the downregulation of TaMYB20-like is still unresolved in our work. Although transcriptome data show that foliar selenium treatment represses TaMYB20-like transcript abundance, the upstream selenium-responsive signaling components mediating this transcriptional inhibition remain unknown. Moreover, we have predicted putative binding sites of TaMYB20-like in the promoters of anthocyanin biosynthetic genes, but biochemical experiments such as EMSA, Y1H, or ChIP-qPCR are still lacking to confirm direct protein–DNA interactions between TaMYB20-like and ANS, DFR, or CHS. Such biochemical validations will be prioritized in our follow-up investigations.

Third, metabolite measurements in this work mainly focused on grain tissues. We did not determine anthocyanin, total phenol, and total flavonoid concentrations in vegetative organs such as leaves. Targeted metabolomics for identifying individual phenolic and anthocyanin compounds was also not performed. Further experiments covering multiple tissue types and targeted metabolomic profiling will help comprehensively dissect selenium-triggered metabolic reprogramming in black wheat.

## 5. Conclusions

Foliar spraying of 75 mg·L^−1^ sodium selenite exhibited optimal comprehensive effects on improving grain selenium accumulation and antioxidant metabolite levels in black-grained wheat cv. Nongda 3753. Selenium-mediated enhancement of anthocyanin and phenolic antioxidant biosynthesis displayed distinct developmental dependence throughout grain filling, with the 25 DAA stage representing a critical responsive window for Se-induced metabolic reprogramming. Transcriptomic analysis revealed that the anthocyanin structural gene ANS was markedly activated by selenium treatment and may function as a key rate-limiting component governing pigment accumulation. A total of nine transcription factors associated with flavonoid metabolism were screened, among which TaMYB20-like showed consistent downregulation under selenium treatment across all filling stages and is proposed to act as a negative regulator of anthocyanin biosynthesis.

## Data Availability

All physiological biochemical measurement data, gene amplification primers, and Supplementary Tables are supplied as Supplementary Materials attached to this article.

## Supplementary Materials

The following supporting information can be downloaded at: https://www.mdpi.com/article/10.3390/plants15182880/s1, Table S1: Primer sequences used for RT-qPCR in this study. Table S2: Summary of Nongda3753 RNA-seq data.Treatment and control groups are designated as SeT-XX and CK-XX, respectively, where “XX” denotes days after anthesis (DAA). Table S3: The functions of DEGs displayed marked stage-specific patterns across developmental stages.
