# Supplementary material for: Effects of Foliar Selenium Treatment on Phenolic Antioxidant Compound Accumulation and Transcriptomic Profiles in Developing Black Wheat Grains
Source: Plants (Basel). 2026 Sep 20;15(18):2880. doi: 10.3390/plants15182880 (PMC13610987; doi:10.3390/plants15182880)
Supplement: Supplementary file 1 [file plants-15-02880-s001.zip › plants-4522325-supplementary.pdf]

Supplemental Table S1 Primer sequences used for qRT-PCR in this study.

Gene	Forward primer (5'-3')	Reverse primer (5'-3')
TaACTIN	TGTTGTTCTCAGTGGAGGTTCT	CTGTATTTCCTTTCAGGTGGTG
LOC123039618	GTACCACCCCGTCCATCATC	TTTTCCGCTCGCTTCAGTCT
LOC123135670	CGCAACCATTTCTGCCACAA	ATTGGTTGTTGTGGTTGCGG
LOC123186676	TGCGTTCCTCTATGCCATCG	CATGCTTGCCGAACTCCAAC
LOC123102968	CATCTCCAGAGCAGCAACCA	AGCTGCTATGTTGCACCACT
LOC100192198	CAACGGCAGGTTCAAGAACG	GCGGTACATCTCGGAGAAGG
LOC123186261	GGATTACCGTCCAGCCCATC	TCTTGAACCTCCCGTTGCTC
LOC100037526	CACCGGGTCATGGTCATCTC	GCAGTGGAAGTACCTCACCC
LOC123098227	ACAGGAGAGGAGGAGGGAGGA	ACCCTCTCCCTTGACCTACC
LOC123166533	CAACAGACACCAACGAAGCG	ATCGTGCCGAAGCTGACGTA
TaACTIN	TGTTGTTCTCAGTGGAGGTTCT	CTGTATTTCCTTTCAGGTGGTG
TaMYB20-like	AGCCGTGCTGCGACAAGGT	GGAGAGTAGCCCCCGCTTGAG
AtMYB20	TCGAGTGGTCCGACTATGGT	TTTTCTGTTTCCCCCACCCC
AtActin2	CTTTGCCGCGAGAACCA	CACTCGAACTCCGGGAACTG
AtMYB20	AGTTGCAGACTTCGTTGGACT	ACCTGTTTCCAAGCTGGGAA
CHS	AATGGTGATGCTGCTGGTCTT	TGGTGATGCGGAAGTAGTAGTC
F3H	CAGGGACGAAGATGAACGGC	AAGCAAAAGAAGTCACGAGCG
DFR	TGGTGTCGGTCCATTCAT	GAGAGAGCGCGGTGATAAGG
UF3GT	TGGAGGTGGCGGTTGAA	GTGCCCGTCTTCATGGAGC

Supplemental Table S2 Summary of Nongda3753 RNA-seq data.Treatment and control groups are designated as SeT-XX and CK-XX, respectively, where "XX" denotes days after anthesis (DAA).

Sample	Raw Reads	Clean Read	Valid Ratio	Q30%	GC content%
CK-5-1	41498332	40652070	97.96%	93.70%	54.09%
CK-5-2	50744488	49806270	98.15%	93.54%	54.42%
CK-5-3	45847710	44326460	96.68%	93.93%	55.29%
SeT-5-1	47977550	47296272	98.58%	94.44%	53.68%
SeT-5-2	44451156	43806296	98.55%	94.07%	53.66%
SeT-5-3	46517492	45734892	98.32%	93.61%	54.22%
CK-15-1	45473086	44105660	96.99%	94.86%	51.16%
CK-15-2	40368912	39106038	96.87%	94.90%	51.89%
CK-15-3	42521512	41238100	96.98%	94.71%	53.82%
SeT-15-1	39081746	37958924	97.13%	94.13%	52.85%
SeT-15-2	41934580	40667070	96.98%	94.58%	53.04%
SeT-15-3	36365324	35302830	97.08%	93.86%	52.71%
CK-25-1	44920586	43401066	96.62%	93.34%	57.82%
CK-25-2	52303092	50670670	96.88%	94.06%	58.64%
CK-25-3	56164496	54327914	96.73%	93.81%	57.64%
SeT-25-1	48894514	47284878	96.71%	94.29%	58.33%
SeT-25-2	52565394	51028822	97.08%	94.53%	57.86%
SeT-25-3	47996408	46956532	97.83%	93.39%	58.82%
CK-35-1	51541690	50113068	97.23%	94.52%	58.43%
CK-35-2	45366860	44525952	98.15%	93.60%	60.04%
CK-35-3	47114054	46065608	97.77%	93.26%	57.43%
SeT-35-1	46581568	45172750	96.98%	93.63%	59.04%
SeT-35-2	42770674	41317910	96.60%	94.01%	59.82%
SeT-35-3	52860058	51994428	98.36%	94.11%	58.95%
Average	46327553	45119186	97.84%	94.04%	55.99%

Supplemental Table S3 The functions of DEGs displayed marked stage-specific patterns across developmental stages.

Category	Up/Down	BP		CC		MF		Total	
		Number	Proportion	Number	Proportion	Number	Proportion	Number	Proportion
SeT-5 vs CK-5 GO term	Up	311	13.71	322	14.20	265	11.68	898	39.59
	Down	489	21.56	488	21.52	393	17.33	1370	60.41
	Total	800	35.27	810	35.71	658	29.01	2268	100.00
SeT-5 vs CK-5 gene	Up	1507	19.37	1109	14.25	417	5.36	3033	38.98
	Down	2286	29.38	1947	25.02	515	6.62	4748	61.02
	Total	3793	48.75	3056	39.28	932	11.98	7781	100.00
SeT-15 vs CK-15 GO term	Up	809	30.39	766	28.78	656	24.64	2231	83.81
	Down	148	5.56	165	6.20	118	4.43	431	16.19
	Total	957	35.95	931	34.97	774	29.08	2662	100.00
SeT-15 vs CK-15 gene	Up	3731	43.60	2557	29.88	803	9.38	7091	82.87
	Down	684	7.99	591	6.91	191	2.23	1466	17.13
	Total	4415	51.60	3148	36.79	994	11.62	8557	100.00
SeT-25 vs CK-25 GO term	Up	2068	28.36	1911	26.21	1840	25.23	5819	79.80
	Down	529	7.25	524	7.19	420	5.76	1473	20.20
	Total	2597	35.61	2435	33.39	2260	30.99	7292	100.00
SeT-25 vs CK-25 gene	Up	10329	41.89	7017	28.46	2254	9.14	19600	79.48
	Down	2484	10.07	2058	8.35	517	2.10	5059	20.52
	Total	12813	51.96	9075	36.80	2771	11.24	24659	100.00

Supplemental Table S3 (continued)

Category	Up/Down	BP		CC		MF		Total	
		Number	Proportion	Number	Proportion	Number	Proportion	Number	Proportion
SeT-35 vs CK-35 GO term	Up	247	8.72	236	8.33	213	7.52	696	24.56
	Down	740	26.11	763	26.92	635	22.41	2138	75.44
	Total	987	34.83	999	35.25	848	29.92	2834	100.00
SeT-35 vs CK-35 gene	Up	1066	10.84	857	8.72	235	2.39	2158	21.95
	Down	3752	38.16	3051	31.03	870	8.85	7673	78.05
	Total	4818	49.01	3908	39.75	1105	11.24	9831	100.00
